# Label-free quantitative proteomics of *Sorghum bicolor* reveals the proteins strengthening plant defense against insect pest *Chilo partellus*

**DOI:** 10.1186/s12953-021-00173-z

**Published:** 2021-04-02

**Authors:** Vaijayanti A. Tamhane, Surhud S. Sant, Abhilash R. Jadhav, Abdul R. War, Hari C. Sharma, Abdul Jaleel, Akanksha S. Kashikar

**Affiliations:** 1 Institute of Bioinformatics and Biotechnology (IBB), Savitribai Phule Pune University (SPPU), Ganeshkhind Road, Pune, Maharashtra 411 007 India; 2 Present Address: Indian Institute of Science, Education & Research, Dr. Homi Bhaha Road, NCL Colony, Pune, Maharashtra 411008 India; 3 International Crop Research Institute for the Semi-Arid -Tropics (ICRISAT), Patancheru, Telangana 502324 India; 4 Present Address: World Vegetable Center, South Asia, ICRISAT campus, Patancheru, Telangana 502324 India; 5 Present Address: Dr. YSP University of Horticulture and Forestry, Nauni, Solan, HP 173230 India; 6 Rajiv Gandhi Centre for Biotechnology, Thiruvananthapuram, Kerala 695014 India; 7 Department of Statistics, SPPU, Ganeshkhind Road, Pune, Maharashtra 411 007 India

**Keywords:** *Chilo partellus*, Insect pests, *in-solution* proteomics, Plant defense, Label-free quantitative proteomics

## Abstract

**Background:**

Spotted stem borer- *Chilo partellus* - a Lepidopteran insect pest of *Sorghum bicolor* is responsible for major economic losses. It is an oligophagous pest, which bores through the plant stem, causing ‘deadheart’ and hampering the development of the main cob. We applied a label-free quantitative proteomics approach on three genotypes of *S. bicolor* with differential resistance/ susceptibility to insect pests, intending to identify the *S. bicolor’s* systemic protein complement contributing to *C. partellus* tolerance.

**Methods:**

The proteomes of *S. bicolor* with variable resistance to insect pests, ICSV700, IS2205 (resistant) and Swarna (susceptible) were investigated and compared using label-free quantitative proteomics to identify putative leaf proteins contributing to resistance to *C. partellus*.

**Results:**

The multivariate analysis on a total of 967 proteins led to the identification of proteins correlating with insect resistance/susceptibility of *S. bicolor*. Upon *C. partellus* infestation *S. bicolor* responded by suppression of protein and amino acid biosynthesis, and induction of proteins involved in maintaining photosynthesis and responding to stresses. The gene ontology analysis revealed that *C. partellus*-responsive proteins in resistant *S. bicolor* genotypes were mainly involved in stress and defense, small molecule biosynthesis, amino acid metabolism, catalytic and translation regulation activities. At steady-state, the resistant *S. bicolor* genotypes displayed at least two-fold higher numbers of unique proteins than the susceptible genotype Swarna, mostly involved in catalytic activities. Gene expression analysis of selected candidates was performed on *S. bicolor* by artificial induction to mimic *C. partellus* infestation.

**Conclusion:**

The collection of identified proteins differentially expressed in resistant *S. bicolor*, are interesting candidates for further elucidation of their role in defense against insect pests.

**Supplementary Information:**

The online version contains supplementary material available at 10.1186/s12953-021-00173-z.

## Background

*S. bicolor* (L.) Moench is an important food, forage and biofuel Saccharinae crop cultivated world over, and recognized for its high yield and stress tolerance. It is the fifth most important cereal crop in the world after rice, wheat, maize and barley and it is the third important cereal crop after rice and wheat in India [1]. The molecular, biochemical and biotechnological investigations in *S. bicolor* are vital for its sustainable supply and it has been recognized as a model plant system for stress proteomics and genomics research [2, 3]. Over 150 insect species are known to cause damage to *S. bicolor* crops, of which, shoot fly (*Atherigona soccata*), spotted stem borer (*Chilo partellus*), midge (*Contarinia sorghicola*) and head bugs (*Calocoris angustatus, Eurtystylus *spp.) are the major pests. The lepidopteran insect pest *C. partellus* is an oligophagous pest, which feeds on cereals like maize, *S. bicolor*, or other wild grasses and is predominant in the warmer regions of the tropics [4]. Of the 58 species in the *Chilo* genus, *C. partellus* is recognized as a major pest causing estimated global losses of over $300 million annually [5, 6]. *C. partellus* neonates feed on tender leaves, causing leaf-scarification, shot-holes and later bore into the stem, causing deadheart [7], destruction of the meristem, and disruption of flowering/seed set [8, 9].

Crop plants have lost the evolutionarily acquired defense mechanisms, due to domestication and repeated selections for agronomic traits [10]; while insects have expanded their geographical horizons to emerge as pests [11]. In *S. bicolor* breeding programs, studies have emphasized the importance of wild germplasm and host plant resistance as a source of insect defense traits for selection breeding [12, 13]. ‘Omics’ approaches have accelerated the elucidation of regulatory processes, novel molecular mechanisms and adaptations in plant-insect interactions, the findings from which have great potential to steer biotic and abiotic stress tolerance in crop plants [14]. Proteome regulates plant phenotype, its responses to stresses and is intricately linked to its transcriptome and metabolome [15]. Proteomics, with the advances in mass spectrometry, has the promise to provide a snapshot into the molecular and functional networks operating within plants and displays a ‘plant molecular phenotype’ [16].

Proteomic studies in *S. bicolor* are swiftly increasing and are focused mainly on osmotic stress [17], grain development and nutritional quality [18], seed storage protein kafirin accumulation [19], salt tolerance [20], heavy metal tolerance [21, 22], albino mutant [23, 24] and drought tolerance [25, 26]. However, the global proteome analysis of *S. bicolor* insect-resistant genotypes and the genetic, biochemical and molecular mechanisms involved in plant defense against pests is not well elucidated. *S. bicolor* like many cereal crops is heavily sprayed with pesticides during its growth to maintain yields /grain quality [27]. Insights from plant-insect interaction studies will be valuable to envisage and employ the much desired sustainable and environmentally gracious cultivation of *S. bicolor*. *S. bicolor* is known to induce cyanogenic glucoside- dhurrin, toxic cyanides and other secondary metabolites such as triterpenols upon insect infestation [28]. Genes like NBS LRR and disease resistance phloem protein 2 were identified as contributors of defense against the sugarcane aphid *Melanaphis sacchari* [29], however, omics and molecular studies on lepidopteran pests of *S. bicolor* are scarce.

*S. bicolor*– lepidopteran insect pest interaction proteomics has been attempted in this study to identify the proteins contributing to insect defense in three sorghum genotypes with varied susceptibility to the spotted stem borer infestation. *S. bicolor* genotypes ICSV700 and IS2205 are known to have variable degree of resistance to *C. partellus* respectively [1, 30] while the cultivated variety (Swarna) is susceptible. The genotypes were evaluated for insect resistance based on percentage of a ‘deadheart’ formation, the extent of leaf damage, stem tunneling, panicle damage and recovery [30].

The proteomics of leaves of *S. bicolor* genotypes at steady-state and upon infestation by the stem borer *C. partellus* has been performed with an objective to (i) elucidate the important proteins contributing to *S. bicolor* insect resistance/susceptibility (ii) proteome complement specific to *S. bicolor* genotype and *C. partellus* treatment. Thorough multivariate statistical analyses for simultaneous comparisons across more than two groups were performed on the proteomics data using the open-source statistical software R. The identified proteins need to be evaluated for potential to enhance plant defense against insect pests and will be useful to engineer these traits to improve sustainable insect tolerance in *S. bicolor*.

## Materials and methods

### Plant material and treatments

**Fig. 1:**
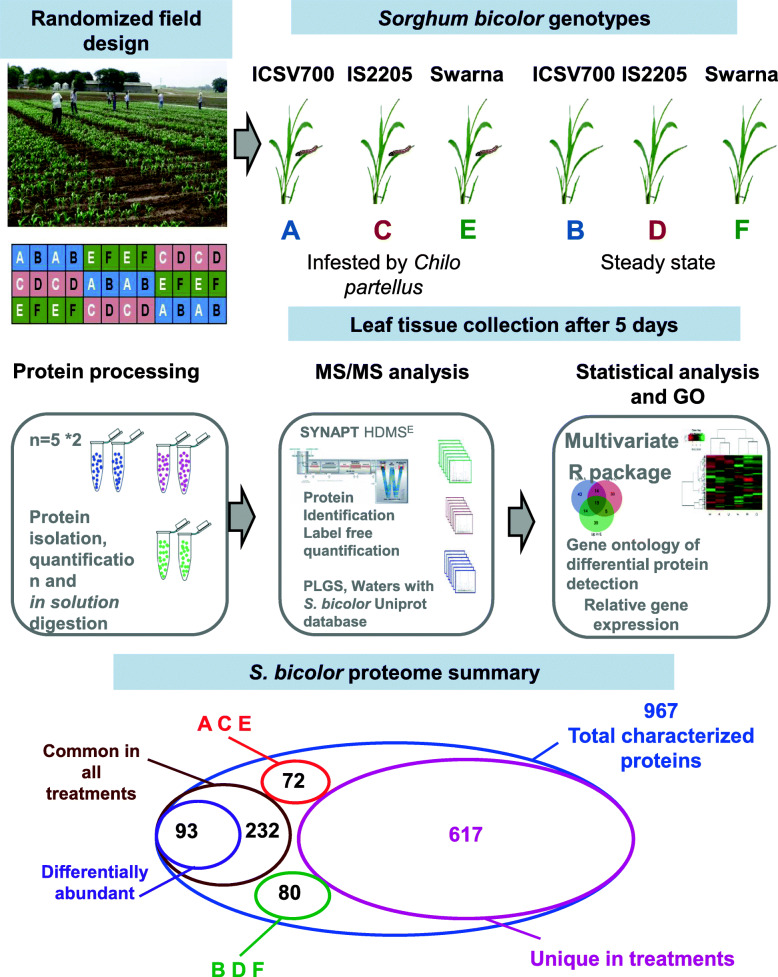
Experimental overview| Three *S. bicolor* genotypes (ICSV700, IS2205, Swarna) with varied insect-susceptibility were planted in the field in randomized block design. Insect-infestation was carried out with the Bazooka applicator and leaves were collected 5 days post-infestation. Leaves from 5 plants were pooled and considered as a biological replicate, and two such replicates were used in the analysis. The proteins were isolated from leaves and subjected to *in-solution* digestion. The MS-MS analysis was performed with SYNAPT HDMS^E^ and *S. bicolor* proteome was used for protein identification. Proteins were analyzed using non-parametric multivariate tests using R. Further, gene ontology and gene expression analysis of proteins were performed

Three *S. bicolor* genotypes, two resistant (ICSV700, IS2205) and one cultivated, susceptible (Swarna) to infestation by insect pest *C. partellus* were grown in the fields at the International Crop Research Institute for the Semi-Arid Tropics (ICRISAT), Patancheru, India (Table 1). Plants were grown in a randomized complete block design (RBD) (Fig. 1) containing 4-row plots of 2 m length, with ridges 75 cm apart. The seedlings were thinned and the planting was maintained at 20 seedlings per 2 m row. The infestation with *C. partellus* was carried out in fields 18 days after germination with the help of the Bazooka applicator [5]. Un-infested rows were maintained as a control. Young leaves (5–8 g) from insect-infested and the un-infested (control) plants were collected 5 days post infestation and flash-frozen in liquid nitrogen. It has been reported that plants signal defense against insect pests at a local level, in plant tissue damaged by the insect as well as at the systemic level, in an undamaged part of the plant [31–33]. Leaves represent a systemic tissue of *C. partellus* infested *S. bicolor* plants as the actual feeding by insect happens at the leaf bases and in the stem. Leaves collected from five plants were pooled and considered as a biological replicate, and two such replicates were collected per treatment. This was done for all the three *S. bicolor* genotypes with *C. partellus* infestation (A, C, E) and control (steady-state) (B, D, F) treatments as abbreviated and detailed in Table 2.

**Table 1:** Characteristics of *S. bicolor* genotypes used in the proteomics study

Characteristics	***S. bicolor*** genotypes		
	ICSV700	IS2205	Swarna
**Panicle**	Fully exerted, compact, elliptic and presence of awns.	Semi-compact and elliptic. Panicle weight of 53 g.	Fully exerted, loose, erect and absence of awns.
**Flowering**	It takes 80–85 days to flower and matures in 120–125 days.	Takes about 80 days to flowering, and matures in about 90–100 days.	Flowering takes place after 65 days.
**Grains**	Lustrous, small-sized grains and 55% grain covered with glumes. 100 seeds weigh around 2.3 g.	White, lustrous. 100 seed weight of 2.6 g.	Lustrous and around 25% grains are covered with glumes. Mass of 100 seeds is around 3.5 g.
**Plant height**	250 cm	250 cm	up to 166 cm
**Insect Resistant/ Susceptible**	Moderately Resistant	Resistant	Susceptible

Morphological, growth, seed features and *Chilo partellus* susceptibility of the three *S. bicolor* genotypes used [30]

**Table 2:** Summary of *in solution* proteomics study of leaves of three *S. bicolor* genotypes at steady-state & upon *C. partellus* infestation

Genotype	Sample code	Treatments	Tech. replicates	No. of proteins
**ICSV700 (Resistant)**	**A**	Infested	1	384
			2	291
			3	347
		Infested	1	396
			2	392
			3	388
	**B**	Steady-state	1	538
			2	450
			3	448
		Steady-state	1	367
			2	313
			3	355
**IS2205 (Resistant)**	**C**	Infested	1	426
			2	368
			3	378
		Infested	1	380
			2	359
			3	338
	**D**	Steady-state	1	483
			2	421
			3	425
		Steady-state	1	440
			2	364
			3	312
**Swarna (Susceptible)**	**E**	Infested	1	324
			2	290
			3	298
		Infested	1	370
			2	306
			3	257
	**F**	Steady-state	1	313
			2	332
			3	340
		Steady-state	1	347
			2	327
			3	289

### Insect rearing and artificial infestation

*C. partellus* larvae were obtained from the insect rearing laboratory at the ICRISAT, Patancheru, India. The insects were reared on *S. bicolor-*based semi-synthetic artificial diet under controlled conditions (16:8 h L: D at 25 ± 1 °C and 65 ± 5% RH) as reported [5]. Newly emerged larvae were mixed with poppy seeds and released onto the leaf whorls of 18–20 days old plants by the Bazooka applicator [5]. About 10 larvae were released on each plant using two strokes of the Bazooka.

### Protein extraction, LC-MS/MS and data analysis

Total protein extraction was done using a phenol extraction method as described earlier [34]. In short, *S. bicolor* leaf tissues stored at − 80 °C were ground to a fine powder in liquid nitrogen with mortar and pestle. The total proteins were extracted from the frozen leaf powder (~ 1.5 g) using the phenol extraction method and they were quantified with Bradford assay [35] using Bovine serum albumin (BSA) as a standard. Protein quality was checked by resolving proteins on 12% SDS-PAGE. Proteins were reconstituted to a final concentration of 1 μg/μL with 0.1% Rapigest™ in 50 mM ammonium bicarbonate. One hundred microgram of protein from each sample (1 μg/μL) was used for *in-solution* reduction and alkylation followed by trypsin digestion to obtain the peptides [34].

Peptide samples were analyzed using a nano ACQUITY UPLC chromatographic system (Waters, Manchester, UK) where each sample was run thrice to obtain three technical replicates corresponding to each biological replicate (Table 1). The instrument was operated and controlled by MassLynx4.1 SCN781 software. The peptide resolution conditions were as detailed by Sharan et al [34]. SYNAPT® G2 High Definition MS™ System (HDMS^E^ System) (Waters Corporation, Milford, USA) was used to carry out mass spectrometry analysis of eluting peptides with instrument settings as; nano-ESI capillary voltage – 3.4 kV, sample cone - 40 V, extraction cone - 4 V, IMS gas (N_2_) flow - 90 (ml/min). All analyses were performed using positive mode ESI using a NanoLockSpray™ source as detailed in [34]. Protein identification and label-free relative protein quantification were done by analyzing LC-MS/MS data using ProteinLynx Global Server™ v2.5.3 (PLGS, Waters Corporation) for each technical replicate. Noise reduction thresholds for low energy scan ion, high-energy scan ion, and peptide intensity were set at 150, 50 and 500 counts, respectively as suggested by the manufacturer. A peptide was required to have at least two assigned fragments, and a protein was required to have at least 2 assigned peptides and 3 assigned fragments for identification. *S. bicolor* database downloaded from the UniProt database (http://www.uniprot.org/proteomes/UP000000768; the number of sequences 41,380) was searched for protein identification and the protein false positive rate was set to 4%. A ratio of > 1.5 represented over-represented proteins and < 0.65 represents under-represented proteins (Fig. 3, Supplementary Data 1). The number of proteins identified in each of the biological and technical replicates of the *S. bicolor* genotypes is reported in Table 1.

### *In house* statistical analysis of the proteomics data

**Fig. 2:**
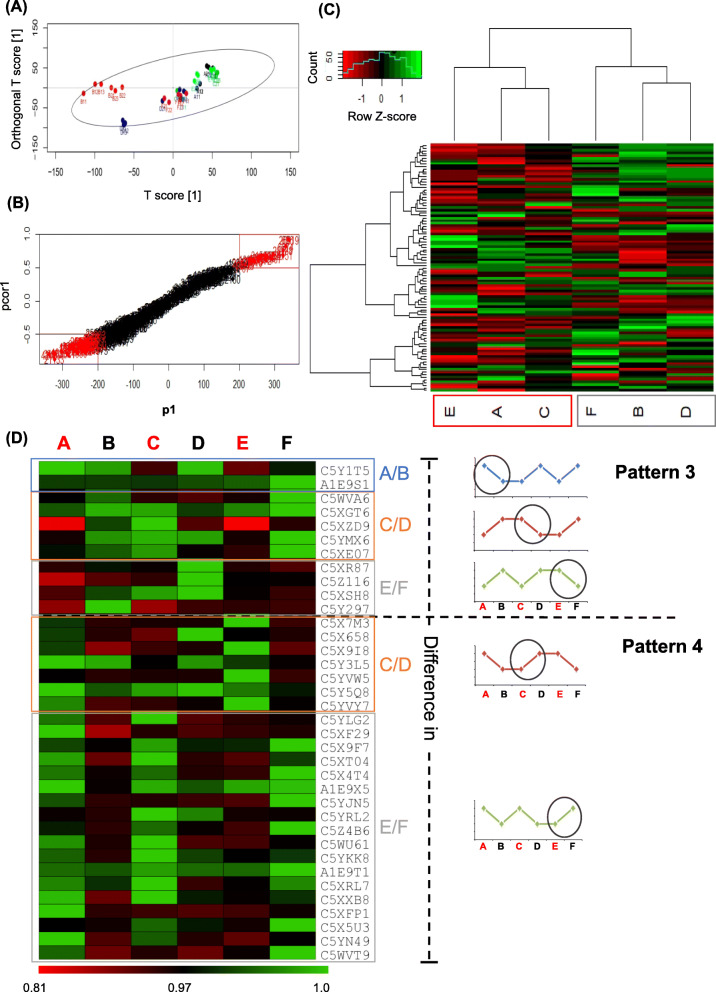
Statistical multivariate analysis of *S. bicolor*- *C. partellus* proteomics study| (**A**) OPLS-DA score plot, indicates that the induced sorghum varieties tend to have positive values of T score as well as orthogonal T score (except for C1), whereas the un-induced varieties tend to have negative values of orthogonal T score (except for B2) (**B**) The S-plot obtained from OPLS-DA helps in identifying most significantly differentially expressed proteins in *S. bicolor* genotypes at steady-state and upon *C. partellus* infestation. (**C)** Clustering analysis was used to identify closeness in protein abundance and indicated the distinct signatures between treatments steady-state and *C. partellus* induced *S. bicolor* genotypes. Heat-map shows the variation in the protein expression across *S. bicolor* genotypes at steady-state and upon *C. partellus* infestation. (**D)** Heat map showing expression levels of proteins common yet differential amongst *S. bicolor* genotypes under various treatments - (A**-F**). Proteins from Pattern3 and Pattern4 have been normalized across rows and each row gives information about a single protein abundance indicated by the UniProt accession number on right. Proteins expressed in these patterns are differentially abundant (over-represented or under-represented) across insect-infested and steady-state comparisons. Graphs on the right, represent expression patterns of the proteins across treatments in resistant and susceptible *S. bicolor* genotypes

Proteomics data from the *S. bicolor* genotypes at steady state and upon *in field C. partellus* infestation (consisting of two biological replicates per treatment with three technical replicates each) was analyzed using multiple non-parametric statistical tests. The pipeline used for analysis was developed *in-house* using R (https://www.R-project.org/) for comparing multiple treatments simultaneously. Considering the biological and technical runs samples (A-F) was represented by six replicates each. Proteins found in at least two technical replicates were considered as truly present and were used for further analysis. The protein data along with the intensity values were log-transformed with base 2 and median normalization was carried out to remove the effect of outliers. Kruskal-Wallis test (for multiple groups) was used instead of ANOVA to compare the results among the samples as it is more robust, can handle an unequal number of observations and non-parametric method that works better for small sample sizes. The *p*-values were adjusted to control the false discovery rate at 5%. Multivariate statistical techniques viz. Cluster Analysis, Principal Component Analysis (PCA) and Orthogonal Partial Least Squares Discriminant Analysis (OPLS-DA) were used to study the similarities and differences among protein expression patterns from different samples (Fig. 2). An average of all technical and biological replicates was used to avoid the problem of missing values during cluster analysis. As a result, for each protein, we had only six readings, one corresponding to each treatment.

In PCA, proteins identified from each technical replicate were used independently. The missing values were replaced by zeros. Proteins showing significantly different abundance from both ends of the S-plot were identified (in all 68 proteins) and studied separately to examine their behavior in each of the six groups (Table 3). The proteins commonly found in all treatments were subjected to pair-wise comparisons using the Mann-Whitney test (a non-parametric equivalent of the t-test, which can handle an unequal number of observations), to identify the proteins which were differentially expressed in either susceptible/resistant or induced/un-induced samples. Proteins not commonly found across samples (A-F) were further studied in the following ways (i) proteins uniquely present in an individual sample, (ii) proteins common in *C. partellus* induced *S. bicolor* were studied as ACE comparison group and (iii) proteins common in the steady-state samples were studied as (iii) BDF comparison group represented in (Fig. 4). In the case of the infested group (ACE) and un-infested group (BDF), averaged out log-transformed data for each protein from all technical replicates was used to generate a normalized (across comparison groups) heat map using MeV 4.9.0 Multiple Experiment Viewer [36].

**Table 3:** Differentially abundant *S. bicolor* proteins identified from the S-plot analysis of the *in solution* proteomics data

Status	Protein Key	Protein Accession No.	Name of protein/similar protein	Function/ GO
Up	372	C5X1U2	Calmodulin	Calcium ion binding (GO:0005509), calcium-mediated signaling (GO:0019722)
Up	656	C5YSK7	similar to Pathogenesis related protein 5	Defense response (GO:0006952)
Up	1292	C5YBE9	Chitin-binding type-1 domain-containing protein	Chitinase activity (GO:0004568)
Up	1510	C5YSK6	similar to Thaumatin like pathogenesis related protein 1	Defense response (GO:0006952)
Up	5767	C5Z0N8	Peroxidase	2 phenolic donor + H2O2 = 2 phenolic radical donor + 2 H2
Up	6674	C5XHS1	similar to β-1,3-glucanase	Hydrolysis of O-glycosyl compounds, Carbohydrate metabolic process
Up	9254	C5XCE2	similar to Zeamatin-like protein	Inhibition of trypsin and α-amylases, Defense response (GO:0006952)
Up	9604	C5Z469	Peroxidase	2 phenolic donor + H2O2 = 2 phenolic radical donor + 2 H2O
Up	10,330	C5WZ07	similar to Glutathione S-transferase	Glutathione transferase activity (GO:0004364)
Up	11,895	C5YSV2	similar to Thaumatin like pathogenesis related protein 5	Defense response (GO:0006952)
Up	12,145	C5YYT5	similar to 60S acidic ribosomal protein P2B isoform X1	
Up	13,645	C5Z3A0	SCP domain-containing protein	similar to pathogenesis-related protein
Up	14,437	C5WWX5	similar to Histone2A	
Up	17,199	C5Z9A2	similar to Thylakoid lumenal 16.5 kDa protein	Photosystem II repair (GO:0010206)
Up	19,206	C5WT31	similar to DPP6 N-terminal domain-like protein	
Up	23,877	C5YLY5	similar to Ribosome-recycling factor	
Up	26,619	C5Y817	similar to Carboxyl terminal peptidase precursor	Peptidase activity
Up	26,971	C5X8S2	SCP domain-containing protein	Cysteine rich secretory protein, allergen V5/Tpx-1
Up	28,788	C5WQE1	similar to α-amylase/ trypsin inhibitor	
Up	30,151	C5Z8N5	Expansin-like EG45 domain-containing protein	Chitinase activity
Up	31,567	C5YGE3	similar to Abscisic acid stress ripening 3	
Up	31,569	C5Y5D6	Barwin domain-containing protein	Defense response to bacterium (GO:0042742) or fungus (GO:0050832)
Down	34	A1E9V4	Cytochrome b6	Component of the cytochrome b6-f complex
Down	102	A1E9W6	50S ribosomal protein L2, chloroplastic	Mitochondrial translation (GO:0032543)
Down	121	A1E9W0	30S ribosomal protein S8, chloroplastic	Translation (GO:0006412)
Down	260	C5YH12	Caffeic acid O-methyltransferase	Flavonol biosynthetic process (GO:0051555)
Down	353	C5XYX5	similar to 60S ribosomal protein L11–1	Translation (GO:0006412)
Down	1163	C5X1Q1	similar to Hydroxyproline-rich glycoprotein family protein	
Down	1442	C5Y065	Lipase_3 domain-containing protein	Lipid metabolic process (GO:0006629)
Down	1979	C5YIF8	Obg-like ATPase 1	ATPase activity (GO:00016887), Negative regulation of response to salt stress (GO:1901001)& defense response to bacterium (GO:1900425)
Down	3699	C5YRK9	similar to Pentatricopeptide repeat-containing protein	RNA modification (GO:0009451)
Down	4242	C5XW30	similar to Phorphobilinogen deaminase	It catalyzes head to tail condensation of four porphobilinogen molecules releasing 4 ammonia molecules
Down	5841	C5YRL0	Non-specific lipid transfer protein	Bifunctional protease and alpha amylase inhibitor inhibitor, lipid binding (GO:0008289) lipid transfer (GO:0006869) protein
Down	6172	C5XYT6	FAD_binding_3 domain-containing protein	FAD binding (GO:0071949),Geranylgeranyl reductase activity (GO:0045550)
Down	10,362	C5YL07	Aldedh domain-containing protein	Betaine-aldehyde dehydrogenase activity (GO:0008802), Response to anoxia (GO:0071454)
Down	11,647	C5WTC9	Ribosomal_L16 domain-containing protein	Translation (GO:0006412)
Down	12,657	C5Z267	similar to 60S ribosomal protein L9	Cytoplasmic translation (GO:0002181)
Down	14,425	C5YAD0	similar to 60S ribosomal protein L6	Cytoplasmic translation (GO:0002181)
Down	15,418	C5XEA1	similar to Fructose-bisphosphate aldolase 1, chloroplastic isoform X1	
Down	15,466	C5YHF2	similar to Rubredoxin family protein	
Down	15,661	C5XZ84	40S ribosomal protein S8	Translation (GO:0006412)
Down	15,716	C5WZ25	Tubulin beta chain	GTPase activity (GO:0003924), microtubule cytskeletal organization (GO:0000226)
Down	16,668	C5YAI8	Pyruvate kinase	ATP + pyruvate = ADP + H+ + phosphoenolpyruvate, Glycolytic process (GO:0006096)
Down	17,564	C5YCD5	PfkB domain-containing protein	Adenosine kinase activity (GO:0004001), Purine ribonucleoside salvage (GO:0006166)
Down	18,075	C5YXW7	Guanosine nucleotide diphosphate dissociation inhibitor	Rab GTPase binding (GO:0017137), small GTPase mediated signal transduction (GO:0007264)
Down	19,332	C5X6V0	similar to Extracellular ribonuclease LE	RNA catabolic process (GO:0006401)
Down	19,346	C5YG66	Aminomethyltransferase	Aminomethyltransferase activity (GO:0004047), Glycine decarboxylation via glycine cleavage system (GO:0019464)
Down	21,133	C5YG29	similar to 60S ribosomal protein	Translation (GO:0006412)
Down	22,396	C5YCD6	Phenylalanine ammonia-lyase	L-phenylalanine = NH4+ + trans-cinnamate, Cinnamic acid biosynthetic process (GO:0009800), L-phenylalanine catabolic process (GO:0006559)
Down	22,977	C5WT26	40S ribosomal protein S4	Translation (GO:0006412)
Down	23,733	C5YX57	40S ribosomal protein S4	Translation (GO:0006412)
Down	23,995	C5YU66	similar to Heat shock 70 kDa protein 4	Stress response
Down	24,630	C5YJP1	HATPase_c domain-containing protein	Unfolded protein binding (GO:0051082), Response to chlorate (GO:0010157), heat (GO:0009408), salt stress (GO:0009651), water deprivation (GO:0009414)
Down	25,743	C5X255	similar to Formate tetrahydrofolate ligase	
Down	25,986	C5WXD2	similar to Protein TIC110, chloroplastic	
Down	26,465	C5XXT8	Phenylalanine ammonia-lyase	L-phenylalanine = NH4+ + trans-cinnamate, Cinnamic acid biosynthetic process (GO:0009800), L-phenylalanine catabolic process (GO:0006559)
Down	28,031	C5XIT6	Pectinesterase	[(1 → 4)-α-D-galacturonosyl methyl ester](n) + n H2O = [(1 → 4)-α-D-galacturonosyl](n) + n H+ + n methanol, cell wall modification (GO:0042545)
Down	28,874	C5YMU8	similar to Puromycin-sensitive aminopeptidase	
Down	29,216	C5YPW0	similar to ATP-citrate synthase	ATP binding (GO:0005524)
Down	30,618	C5WZ87	similar to Ribosomal protein S9	Translation (GO:0006412)
Down	30,990	C5XI18	S-adenosylmethionine synthase	ATP + H2O + L-methionine = diphosphate + phosphate + S-adenosyl-L-methionine, S-adenosylmethionine biosynthetic process (GO:0006556)
Down	31,330	C5YNT6	S4 RNA-binding domain-containing protein	Translation (GO:0006412), Positive regulation of translational fidelity (GO:0045903)
Down	31,631	C5WXA8	NADPH-protochlorophyllide oxidoreductase	chlorophyllide a + NADP+ = H+ + NADPH + protochlorophyllide a
Down	31,939	C5WZQ4	similar to 50S ribosomal protein L6	Translation (GO:0006412)
Down	32,283	C5XE18	40S ribosomal protein SA	Cytoplasmic translation (GO:0002181), Translation (GO:0006412)
Down	32,318	C5YFQ2	Ribosomal_S17_N domain-containing protein	Translation (GO:0006412)
Down	32,520	C5X0S2	Uroporphyrinogen decarboxylase	4 H+ + uroporphyrinogen III = 4 CO2 + coproporphyrinogen III, Protoporphyrinogen IX biosynthetic process (GO:0006782)
Down	32,758	C5YS19	SAM_MPBQ_MSBQ_MT domain-containing protein	Methyltransferase activity (GO:0008168)

The OPLS-DA analysis followed by S-plot analysis was carried out to identify proteins from *S. bicolor* genotypes that showed significant differential abundance. The commonly expressed proteins identified from *S. bicolor* genotypes in all the treatments namely steady-state and *C. partellus* induced were considered for this analysis

### GO classification, pathway enrichment analysis

**Fig. 3:**
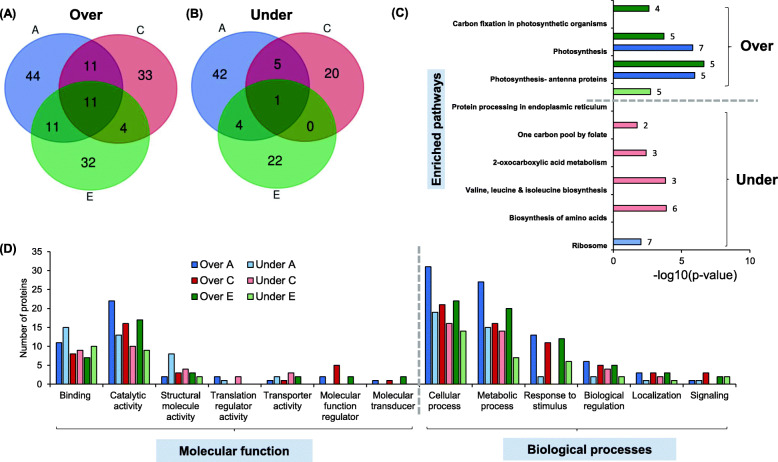
Pathway enrichment and protein-protein interaction analysis of proteins in three genotypes| Venn diagram showing commonly over-represented (> 1.5) (**A**) and under-represented (< 0.65) (**B**) proteins upon *C. partellus* infestation identified from ProteinLynx Global Server™ v2.5.3 (PLGS, Waters Corporation). (**C)** The pathway enrichment analysis of over-represented and under-represented proteins in a particular variety was performed using the g:Profiler web server. The numbers on the right side of each bar graph indicate the number of proteins represented in the enriched pathway. (**D)** The gene ontology analysis of over-represented and under-represented proteins was performed using the PANTHER tool

The functional classification of identified proteins was carried out using the UniProt database [37]. Further, gene ontology (GO) analysis of identified differentially expressed proteins was carried out using the PANTHER tool [38]. Common proteins, unique proteins, proteins from infested and un-infested samples were analyzed for molecular function, biological process and cellular component using accession number as an ID and *S. bicolor* as an organism in the PANTHER tool. Analysis type was selected as functional classification viewed in a pie chart. The pathway enrichment analysis of differentially expressed proteins identified from ProteinLynx Global Server™ v2.5.3 (PLGS, Waters Corporation), was done using g:Profiler web server (Fig. 3C) [39].

### Relative expression profiles of candidates from proteomics data

Poly-house grown, 3 weeks old *S. bicolor* seedlings of - Swarna (susceptible) & ICSV700, IS2205 (resistant) were used for gene expression analysis. *C. partellus* extract prepared in water was applied to mechanically wounded leaves to mimic the insect infestation (W + E). In control samples, wounding was followed by the application of water (W + W) to the leaf. Leaf samples were collected 3 h and 24 h post-treatment. Total RNA was extracted using the Macherey-Nagel NucleoSpin Plant II kit (Macherey Nagel Co., Duren, Germany) according to the manufacturer’s instructions. The concentration of RNA was measured using Nano-Drop (Eppendorf, Biophotometer plus, Hamburg, Germany). The integrity of RNA samples was checked by agarose gel electrophoresis and 2 μg RNA was used for cDNA synthesis using a cDNA synthesis kit (High capacity cDNA Reverse Transcription kit, Applied Biosystems, Foster City, California, United States) as per the manufacturer’s guidelines. Real-time quantitative PCR (7500 Fast real-time PCR systems, Applied Biosystems, Foster City, California, United States) was used to check expression levels of the candidates identified from proteomics analysis using gene-specific primers synthesized at IDT (Coralville, Iowa, United States) (Supplementary Table 3), with the help of GoTaq® qPCR Master Mix (Promega Corporation, Madison, USA). Tubulin was used as a reference house-keeping gene for analysis. The data from 3 biological replicates of leaves were analyzed with 4 technical replicates each. Threshold cycle values (Ct) were used to calculate ΔCt = Ct_Gene of interest_-Ct_Tubulin_ and represented as fold change 2^ΔCt^ in the graphs (Fig. 5). The uninduced control sets for all the 3 genotypes were compared and analyzed using Tukey’s HSD test and indicated by different letters showing significant difference in expression values (Fig. 5). The water treatment (W + W) and insect extract-treated samples (W + E) were compared to the respective controls with the help of a two-tailed Student’s t-test with unequal variance with the threshold of *p < 0.05*.

## Results

### *C. partellus* infestation induces differential shifts in leaf proteomes of three different *S. bicolor* genotypes

The selected *S. bicolor* genotypes namely ICSV700, IS2205 and Swarna varied for their insect susceptibility/resistance and other agronomic traits like plant height, panicle, flowering time, grain characters and grain mass (Table 3). The earlier studies had indicated that ICSV700 and IS2205 were having moderate to good resistance to insect pests respectively, while Swarna was insect susceptible, but displayed desirable agronomic traits namely early flowering, lower plant height and higher seed mass [30]. The leaf proteomics of these three *S. bicolor* genotypes at steady-state (uninduced) and induced with the insect pest *C. partellus* was carried out to identify the *S. bicolor* proteins responsible for insect resistance (Fig. 1). The proteome data consisted of 967 characterized proteins, of which 232 were commonly detected in all treatments, 93 were differentially abundant across treatments, proteins common to a subset of treatments namely -induced A, C, E and steady-state B, D, F were 72 and 80 respectively, while the sum of proteins uniquely detected in each treatment (A-F) were 617. Multivariate analysis of the proteomics data in the form of PCA (Supplementary Fig. 1) and OPLS-DA was performed on all proteins identified in the study. The results indicated the overall distribution of the samples (A-F) and closeness of the biological and technical replicates (except C, D of the *S. bicolor* IS2205) (Fig. 2A). Based on their separation along the X-axis of OPLS-DA (T score) the resistant *S. bicolor* genotype ICSV700 in the uninduced state (B) was strikingly different from the rest of the two. Moreover, upon *C. partellus* induction both the resistant genotypes ICSV700 (A) and IS2205 (C) showed a remarkable proteomic alteration as compared to their corresponding uninduced states (B, D) as indicated by the difference in the T score (Fig. 2A).

The S-plot helped demarcate the overall significantly differential proteins from the *S. bicolor* proteome (Fig. 2B) as detailed in (Table 2). Twenty two proteins from the upper end and 46 from the lower end of the S-plot were identified as significantly differential. Their gene ontology indicated that they were involved in defense and immunity, calcium-binding and signaling, cell wall modifications and catalytic activities; whereas the proteins with less abundance were mostly involved in translation, signaling, and different catalytic activities (Table 2). These proteins may positively or negatively regulate *S. bicolor*’s interaction with *C. partellus* through their involvement in defense, biotic and abiotic stress tolerance, detoxification, enzyme inhibition, hydrolysis activities and signaling.

Cluster analysis was performed on the proteins commonly detected in all the treatments (A-F) (Fig. 2C). The analysis indicated that the proteins from the uninduced *S. bicolor* samples (B, D, F) clustered separately from the *C. partellus* induced samples (A, C, E). Moreover, the insect-resistant *S. bicolor* genotypes namely ICSV700 and IS2205 (represented by A, B and C, D) clustered separately from the insect susceptible *S. bicolor* Swarna (E, F).

Ninety three proteins were found to be differentially expressed in the *S. bicolor* genotypes (A-F), of which 57 proteins displayed similar abundance patterns in the three *S. bicolor* genotypes (Supplementary Fig. 2), representing a fraction of defense response commonly induced by the genotypes upon *C. partellus* infestation. These protein species were further categorized into two patterns- Pattern1 with 38 proteins downregulated upon *C. partellus* infestation and Pattern2 with 19 proteins upregulated upon *C. partellus* infestation in *S. bicolor* genotypes compared to the steady-state (Supplementary Fig. 2). The remaining 36 proteins were important as they were differentially abundant in the resistant and susceptible *S. bicolor* genotypes. They were further grouped into Pattern3 (11 proteins) and Pattern4 (25 proteins) representing under-represented and over-represented proteins in *C. partellus* induced *S. bicolor* respectively, with contrast in protein expression displayed by one of the *S. bicolor* genotypes (Fig. 2D; Supplementary Fig. 3). Pattern3 proteins indicated that the biological process of translation was contrastingly upregulated in resistant *S. bicolor* genotypes. Proteins like Photosystem II subunit, germin-like protein, serine hydroxyl methyltransferase and ATPase alpha subunit were prominent in *C. partellus* induced susceptible Swarna (E) whereas they were under-represented in corresponding treatments of resistant genotypes, ICSV700 (A) and IS2205 (C). In the Pattern4 insect susceptible *S. bicolor* Swarna displayed an under-representation of the proteins which were involved in the biosynthetic process, cellular nitrogen compound process and cellular amino acid metabolism, represented by proteins like glycine-rich protein 2, NAD(P)H-quinone oxidoreductase subunit, profilin-4, Co-chaperone CGE1 isoform b, cysteine synthase, non-specific lipid transfer protein and superoxide dismutase. Ribulose bisphosphate carboxylase, ATP synthase subunit beta, extracellular calcium-sensing receptor and elongation factor 1- delta were up-regulated in the *C. partellus* induced resistant *S. bicolor* genotype IS2205 (C) whereas they were under-represented in the other genotypes.

### Analysis of differential proteins identified in a pairwise comparison of *S. bicolor* genotypes upon *C. partellus* infestation and at steady-state using ProteinLynx global server™ v2.5.3 (PLGS, waters corporation)

Leaf proteomes of *C. partellus* induced and steady states of genotypes of *S. bicolor* were compared with the help of ProteinLynx Global Server™ v2.5.3 (PLGS, Waters Corporation) to identify over-represented (fold change > 1.5) and under-represented (fold change < 0.65) proteins. These proteins were compared to identify proteome similarities/differences amongst the genotypes (Fig. 3, Supplementary Data 1). Most of the differential proteins identified in the pair-wise comparisons were not shared between the 3 genotypes, signifying unique ways of each genotype to deal with the *C. partellus* induction (Fig. 3A & 3B). The enrichment analysis of over-represented proteins from Swarna and ICSV700 is involved in photosynthesis or carbon fixation. Under-represented proteins were enriched for the ribosome, protein processing in the endoplasmic reticulum, biosynthesis of amino acids (Fig. 3C). The gene ontology analysis of these proteins indicated that the majority of them were involved in cellular and metabolic processes related to binding and catalytic activities. It is important to note that *S. bicolor* upon *C. partellus* infestation suppresses the accumulation of several proteins from these GO categories and initiates the accumulation of other proteins representing the same categories (Fig. 3D). Under-representation of proteins related to response to stimulus in Swarna was one interesting find from this analysis. To maximize the useful information derived from the data, the induced and un-induced states were compared separately in further analysis.

### GO analysis of differential proteins in *C. partellus* induced *S. bicolor* (A, C, E) and *S. bicolor* at steady state (B, D, F)

**Fig. 4:**
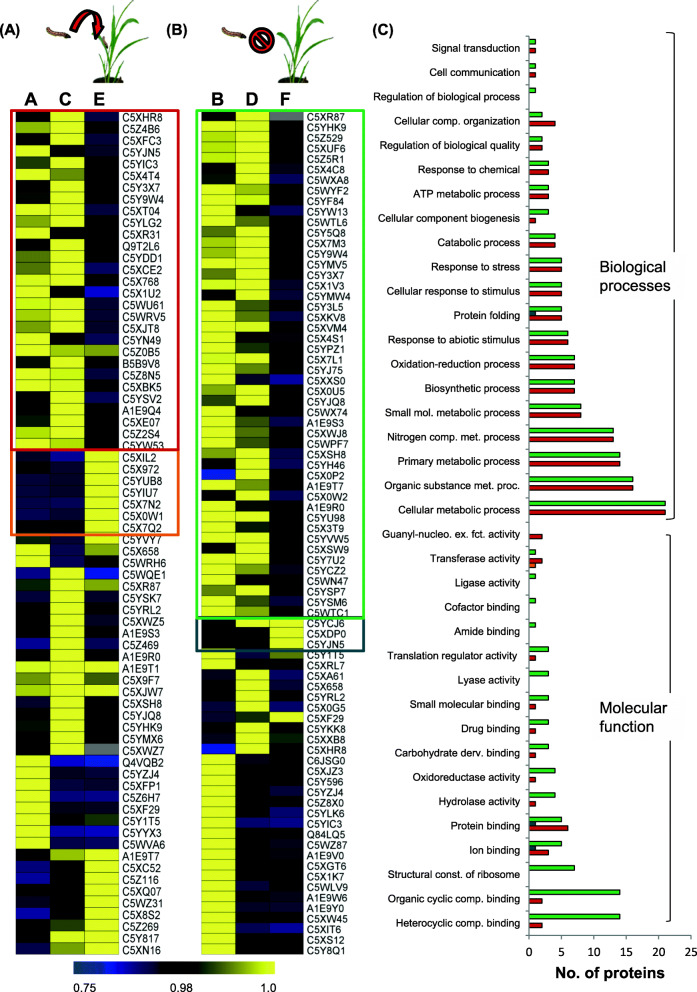
GO analysis, protein abundance of differentially regulated proteins from *C. partellus* infested & steady-state *S. bicolor* genotypes | 72 & 80 proteins were found to be differentially expressed in *S. bicolor* genotypes under treatmetns - A, C, E (**A**) and - B, D, F (**B**) respectively. The heatmap was made by using log-transformed protein expression values normalized across rows. (**A**) Proteins represented with the box were over-represented (orange) and under-represented (red) in susceptible *S. bicolor* Swarna represented by treatment E compared to their levels in resistant genotypes ICSV700 and IS2205 upon *C. partellus* infestation, represented by treatments A and C respectively. (**B**) Proteins represented with the box were over-represented (blue) and under-represented (green) in *S. bicolor* Swarna represented by treatment F,  compared to the other two insect-resistant genotypes at a steady state represented by treatments B and D respectively. (**C**) GO level-2 analysis of proteins exhibiting distinct patterns across resistant & susceptible genotype was indicated in the figure with molecular function, biological processes.

Comparing the insect-induced (A, C, E) or steady-state (B, D, F) treatments across *S. bicolor* genotypes helped to widen the analysis by maximizing the information obtained (Fig. 4). The comparison amongst the three treatments led to the identification of a higher number of differential proteins and also account for the intrinsic differences amongst the varieties. The analysis was done on 72 and 80 proteins differentially abundant in *C. partellus* induced *S. bicolor* genotypes (A, C, E) or at steady state (B, D, F) respectively (Supplementary Table 2 and Fig. 4). Of the set, a large number of protein species were significantly differentially abundant in the susceptible genotype Swarna than resistant genotypes. It represented the protein species through which both the resistant *S. bicolor* genotypes responded similarly to the *C. partellus* infestation. Intriguingly, protein species that were found to be differentially abundant in both the resistant *S. bicolor* genotypes either at steady state or upon *C. partellus* infestation were found to be involved in cellular metabolic processes, organic substance metabolic process, nitrogen compound and small molecule metabolic process, oxidation-reduction and response to abiotic stimuli (Fig. 4C). These proteins had the molecular function (MF) of binding and catalytic activity though these were represented by different proteins in A, C, E or B, D, F comparisons (Supplementary Table 2).

### The *S. bicolor* resistant genotypes are rich in unique proteins

**Fig. 5:**
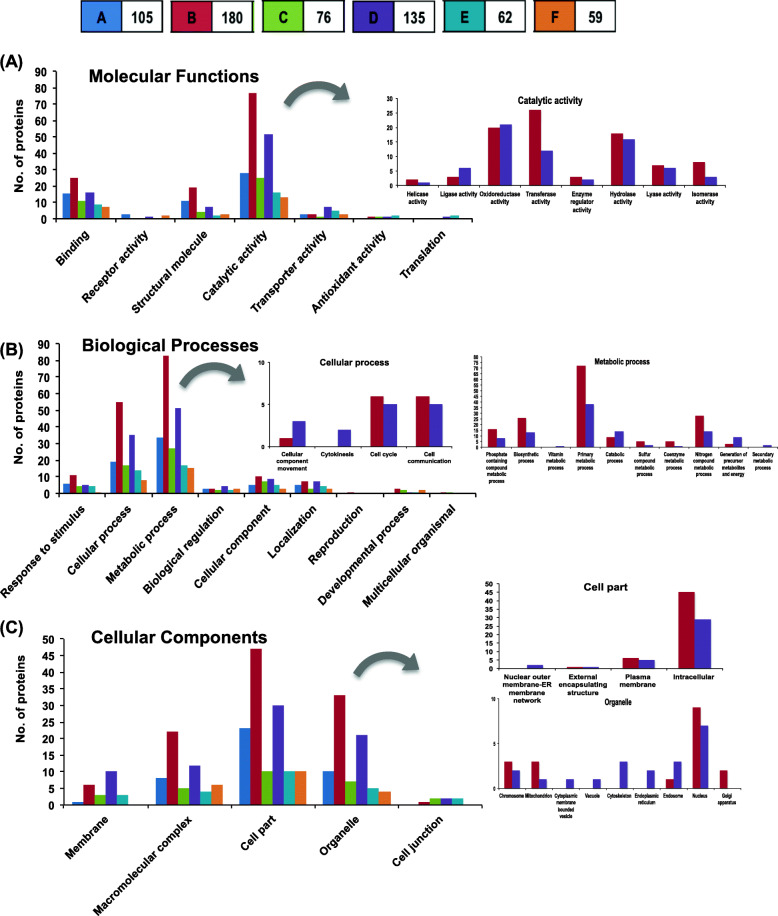
GO analysis of uniquely detected proteins in *S. bicolor* genotypes| The proteomic analysis led to the identification of a large number of uniquely detected proteins in *S. bicolor* genotypes and treatments abbreviated as A, B, C, D, E, F. The horizontal strip with alphabet and number represents treatment and the corresponding number of unique proteins detected. GO analysis of unique proteins involved in molecular function (**A**), biological process (**B**) and cellular component (**C**) have been displayed. The graphs in the insets represent the sub-categories of highly represented proteins

The resistant genotype ICSV700 was found to contain the highest number of unique proteins at steady-state - (B) (180) followed by the other resistant *S. bicolor* IS2205 - (D) (135) while the *C. partellus* induced ICSV700 (A) also displayed around 105 unique proteins (Fig. 5). The GO analysis of the unique proteins identified in each indicated that the molecular functions such as catalytic activity, binding, structural molecular activity were represented predominantly from un-induced resistant genotypes, ICSV700 (B) and IS2205 (D) whereas these functions were very low in the susceptible variety, Swarna. The biological processes like cellular process, metabolic process, cellular component, localization, response to stimulus and cellular components like membrane, macromolecular complex, cell part, organelle were also higher in unique proteins found in un-induced *S. bicolor* resistant genotypes, ICSV700 and IS2205.

The top 10 most abundant unique proteins from each sample (A-F) are listed in Table 4. The *C. partellus* induced ICSV700 (A) showed the presence of proteins like β-caryophyllene synthase involved in indirect defense; RPP-13 like protein, Ankyrin repeat domain-containing protein 2, adenylyl cyclase associated protein which plays an important defense role in plants; proteins involved in protein turnover DNA repair, wound healing was also detected. Some interesting proteins like ATP synthase CF1 alpha subunit involved in inducing changes in plant surface structures like spines were also seen [40]. The other resistant genotype of *S. bicolor* IS2205 (C) upon *C. partellus* infestation showed the unique presence of plant defense proteins like chitinase, RPP-13 like; biotic and abiotic stress-related proteins like monogalactosyldiacyl glycerol synthase, zinc finger CCh domain-containing protein 55, thiazole synthase; and proteins involved in protein turn over. The susceptible *S. bicolor* upon *C. partellus* induction (E), however, showed the expression of proteins like kinases, proteins involved in growth, turnover and homeostasis like adenylate isopentyl transferase, ubiquitin E3-protein ligase, triacylglycerol lipase and UDP d-glucuronate decarboxylase.

**Table 4:** Top 10 of the uniquely represented proteins from *S. bicolor* genotypes at steady-state and upon *C. partellus* infestation

Key	Protein Accession No.	Protein Name	Function
**A -** ***S. bicolor*** **ICSV700 infested by** ***C. partellus***			
23,819	C5Y853	similar to ATP synthase CF1 alpha subunit	Chloroplastic, correlation with spiny-ness
1056	C5WWL7	similar to Beta-caryophyllene synthase	Indirect defense against Lepidoptera by attracting predators
38	C5YUK3	Flap endonuclease 1-A	Catalysis of the cleavage of a 5′ flap structure in DNA, but not other DNA structures; processes the 5′ ends of Okazaki fragments in lagging strand DNA synthesis, Acts as a genome stabilization factor
107	A1E9R4	DNA-directed RNA polymerase subunit beta	DNA-dependent RNA polymerase catalyzes the transcription of DNA into RNA using the four ribonucleoside triphosphates as substrates, Nucleoside triphosphate + RNA(n) = diphosphate + RNA(n + 1)
9314	C5X5B2	similar to ADP-ribosylation factor GTPase-activating protein AGD3	Binds to and increases the activity of a GTPase, plasma membrane remodeling
19,695	C5Y746	similar to disease resistance RPP13-like protein 3 isoform X3	Disease resistance against pathogens
28,942	C5YHK1	similar to Ankyrin repeat domain-containing protein 2	Chloroplast targeting sequence binding
1890	C5XAM0	similar to ubiquitin-like	Protein turnover
20,222	C5X7K7	similar to RNA polymerase beta subunit	RNA polymerization
4393	C5YLQ0	Adenylyl cyclase-associated protein	Cyclase-associated protein 1-like, cytoskeleton organization, response to pathogen
**B -** ***S. bicolor*** **ICSV700 at steady state**			
3162	C5YWC5	similar to Proliferation-associated protein 2G4	Change in state or activity of a cell or an organism as a result of a cytokinin stimulus
28,629	C5Z4X4	similar to reverse transcriptase, Brassinosteroid insensitive-1 like	Plant architecture
7943	C5XJ50	similar to Retrotransposon protein	Probable member of endonuclease, exonuclease, phosphatase family
13,397	C5WSY0	similar to Arginine decarboxylase	Drought tolerance, defense
27,809	C5XAT9	Histone H2A	DNA binding, chromatin silencing
2862	C5XTG6	Nitrate reductase	Cell signaling & survival under stress
11,807	C5WU06	similar to FACT complex subunit SPT16	Histone binding and remodeling outside the context of DNA replication
25,101	C5X957	Ribosomal protein L15	Structural constituent of ribosome, Cytoplasmic translation
14,173	C5WQ44	similar to enolase	Phosphopyruvate hydratase activity
16,161	C5YDV5	similar to putative quinone oxidoreductase	Oxidoreductase activity, chloroplastic
**C -** ***S. bicolor*** **IS2205 infested by** ***C. partellus***			
29,614	C5YIU1	similar to Monogalactosyldiacyl glycerol synthase 2	Thylakoid membrane biogenesis under stress
13,788	C5YMZ5	similar to Zinc finger CCCH domain-containing protein 55-like	ABA biosynthesis, drought, post-transcriptional regulation of gene expression
9243	C5WNH3	similar to ATP binding protein	Protein kinase activity, Serine/Threonine protein kinase STY46 like
5008	C5YGI4	similar to thiazole synthase	ADP binding, Cell wall integrity, and stress response component 1-like
25,363	C5YJ73	similar to Ubiquitin and WLM domain-containing protein	Ubiquitin and WLM domain-containing metalloprotease
5014	C5XXC0	similar to Protein kinase domain-containing protein	Triggered in response to the presence of a foreign body or the occurrence of an injury, Introducing a phosphate group on to a protein, ATP binding, Cysteine-rich receptor-like protein kinase 26
702	C5X8K4	similar to disease-resistant protein RPP-13 like 1	Disease resistance protein against pathogen
17,789	C5Z5B4	similar to 26S protease regulatory subunit 6A-like protein	ATP binding, Interacting selectively and non-covalently with a member of the class of TATA-binding proteins (TBP), including any of the TBP-related factors (TRFs), 26S protease regulatory subunit 6A homolog
19,253	C5YVH3	60S acidic ribosomal protein P0	Ribosomal subunit rRNA binding, Cytoplasmic translation
21,882	C6JSV0	similar to Chitinase	Catalysis of the hydrolysis of (1- > 4)-beta linkages of N-acetyl-D-glucosamine (GlcNAc) polymers of chitin and chitodextrins
**D -** ***S. bicolor*** **IS2205 at steady state**			
28,234	C5Y227	similar to Indole-3-acetic acid-amido synthetase GH3.3	Synthesis of IAA-conjugates, a mechanism to cope up with excess auxin
22,121	C5X8X8	similar to AT-hook motif-containing protein, Helicase	NTP + H2O = NDP + phosphate, to drive the unwinding of a DNA helix, Process of restoring DNA after damage, Telomere maintenance, ATP-dependent DNA helicase PIF1-like
125	C5XNN6	Thiamine thiazole synthase 1, chloroplastic	Involved in the biosynthesis of the thiamine precursor thiazole, Suicide enzyme, Additional roles in adaptation to various stress conditions and DNA damage tolerance
6474	C5WWV5	similar to Inactive ubiquitin carboxyl-terminal hydrolase 53	Thiol-dependent ubiquitinyl hydrolase activity, protein deubiquitination, inactive ubiquitin carboxyl-terminal hydrolase 53
16,964	C5YS29	similar to Diaminopimelate decarboxylase	Diaminopimelate decarboxylase activity, meso-2,6-diaminopimelate + H(+) = L-lysine + CO(2), systemic acquires resistance
31,822	C5XSW5	Glutaredoxin-like protein	Photooxidative stress, antioxidant activity
19,098	C5Z949	similar to RING zinc finger domain superfamily protein	Ubiquitin specific protease binding, ERAD-associated E3 ubiquitin-protein ligase HRD1-like isoform X1
2715	C5XIX0	similar to NEFA-interacting nuclear protein NIP30	Protein FAM192A isoform X1
23,386	C5Y1Y1	Peroxidase	2 phenolic donor + H_2_O_2_ = 2 phenoxyl radical of the donor + 2 H_2_O
29,401	C5Z7K8	Pyruvate dehydrogenase E1 component subunit alpha	Catalyzes the overall conversion of pyruvate to acetyl-CoA and CO_2_
**E -** ***S. bicolor*** **Swarna infested by** ***C. partellus***			
2587	C5XAW9	Serine/threonine-protein kinase	ATP + a protein = ADP + a phosphoprotein, reactions triggered in prevention/recovery from the infection caused by the attack
21,100	C5XLE9	similar to Photosystem II CP47 reaction center protein	Chlorophyll-binding, Photosynthetic ETS, Similar to Photosystem II CP47 chlorophyll apoprotein
21,351	C5XXY1	similar to Serine-threonine kinase receptor-associated protein	Involved in defense
13,794	C5YV23	similar to Adenylate isopentenyl transferase-like	Cytokinin biosynthesis
23,585	C5WW05	similar to Triacylglycerol lipase SDP1	Hydrolase activity, Catalysis of the reaction: triacylglycerol + H_2_O = diacylglycerol + a carboxylate, membrane protein homeostasis
17,550	C5YTB0	similar to Inosine-5′-monophosphate dehydrogenase, Kinesin-like protein	Serine/Threonine Kinase activity
6258	C5YWV3	similar to UDP-D-glucuronate decarboxylase	
349	C5XT35	NADP-dependent D-sorbitol-6-phosphate dehydrogenase	Oxidoreductase activity, sorbitol metabolism, development
28,758	C5Y3U1	similar to BOI-related E3 ubiquitin-protein ligase 1	Abiotic stress tolerance, protein turnover
13,628	C5YHS5	similar to 5′-methylthioadenosine/S-adenosylhomocysteine nucleosidase 2	Catalytic activity, nucleoside metabolic process
**F -** ***S. bicolor*** **Swarna at steady state**			
17,459	C5WPC8	similar to MAR-binding protein	The nuclear envelope protein, development
18,191	C5Y2G1	similar to Filamin B like protein	Connects cell membrane constituents to actin filaments
23,144	C5X4Q7	Histone H2B	DNA binding, Nucleosome assembly
30,485	C5XZI6	similar to B-cell receptor-associated protein 31-like containing protein	ER to Golgi vesicle-mediated transport, Intracellular protein transport, B cell receptor-associated protein 31
4299	C5YXD6	similar to Retrotransposon protein	Nucleic acid-binding, zinc ion binding, bZIP like protein
16,841	C5YBM1	Carboxypeptidase	Serine-type carboxypeptidase activity
7517	C5Z7H3	similar to Putative pentatricopeptide repeat-containing protein	Zinc ion binding, RNA binding, splicing
14,188	C5X6D0	Lon protease homolog, mitochondrial	ATP-dependent serine protease that mediates the selective degradation of misfolded, unassembled or oxidatively damaged polypeptides as well as certain short-lived regulatory proteins in the mitochondrial matrix, protein homeostasis
21,520	C5XXE4	Similar to the nuclear mitotic apparatus protein 1	Mitosis
2898	C5Y2Y9	similar to Clathrin heavy chain 1	Present in a coat of vesicles

Many proteins were found to be uniquely accumulated in specific genotypes and treatments. The top 10 of these unique proteins were selected based on their intensity values obtained from the *in solution* proteomics. The table provides the details of the proteins and their functional significance

The resistant *S. bicolor* genotypes ICSV700 and IS2205, at the steady-state level (B, D) and upon *C. partellus* infestation (A, C) had a far high number of unique proteins while susceptible *S. bicolor* Swarna displayed strikingly smaller number of unique proteins. The susceptible *S. bicolor* variety Swarna lacks the proteins involved in metabolic processes related to nitrogenous compounds, sulfur compounds, secondary metabolites and biosynthetic processes and after infestation by *C. partellus*, it is inefficient in the upregulation of nitrogen compound biosynthesis.

### Relative expression profiles of genes corresponding to protein candidates identified in *S. bicolor*-*C. partellus* interaction proteomics

Serine hydroxymethyltransferase, germins, cyanate hydratase, β-glucanases, lipid transfer proteins (LTP), zeamatin like proteins, endochitinases, superoxide dismutase (SOD), chaperonins and 14–3-3 like proteins were selected for gene expression analysis based on their protein expression pattern in non-targeted *S. bicolor* proteomics study as well as their function. We set up an independent experiment (methods section 2.6) to study the candidate gene expression kinetics at early time points (3 h, 24 h) after mimicking insect infestation.

**Fig. 6:**
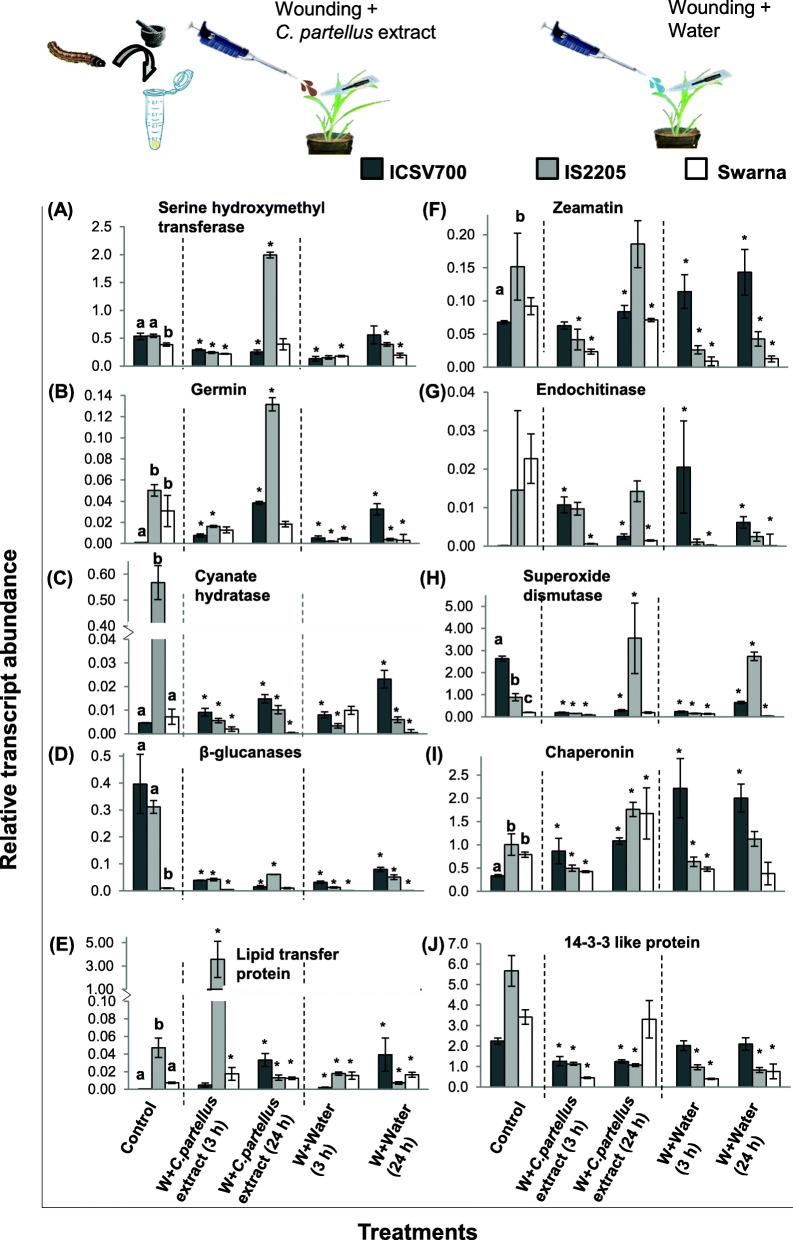
Early (3 & 24 h) gene expression profiles of selected protein candidates from *S. bicolor*-*C. partellus* interaction proteomics study| Relative expression (ΔCt = Ct_Gene of interest_-Ct_Tubulin_) profile of genes corresponding to protein candidates identified from the proteomics study of *S. bicolor* genotypes ICSV700, IS2205 and Swarna at steady-state and after 3 h and 24 h post induced wounding (W + W) and wounding+ *C. partellus* extract (W + E) treatment, have been represented. The relative gene expression profiles for serine hydroxymethyltransferase (**A**), germin (**B**), cyanate hydratase (**C**), β- glucanase (**D**), lipid transfer protein (**E**), zeamatin (**F**), endochitinase (**G**), superoxide dismutase (**H**), chaperonin (**I**), 14–3-3 like protein (**J**) are shown. Tubulin was used as housekeeping control. The student’s t-test followed by Tukey’s HSD (Honestly Significant Difference) test was performed on the data to identify significant differences if any at *p* < 0.05. (*p < 0.05)

The gene expression studies were carried out in the three genotypes of *S. bicolor* (ICSV700, IS2205 and Swarna) under treatments namely (i) steady-state, (ii) wounding + *C. partellus* extract application (W + E) and (iii) wounding + water application (W + W) at 3 h and 24 h post-treatment (Fig. 6). *C. partellus* extract application on the wounded leaf was done to mimic the insect herbivory on *S. bicolor* plants grown in the polyhouse. Distinct gene expression patterns were noted amongst the *S. bicolor* genotypes at steady state. Additionally, the W + E and W + W treatments also displayed differential gene expression patterns at 3 h and 24 h post-treatment across the *S. bicolor* genotypes. ICSV700 showed over-expression of germins, cyanate hydratase, LTP, zeamatin, endochitinase, chaperonins in W + E; whereas serine hydroxymethyltransferases, β- glucanase, SOD, 14–3-3 like proteins were under-expressed in W + E. In W + E, IS2205 genotype showed over-expression of serine hydroxymethyltransferases, germins, SOD, chaperonins and downregulation of cyanate hydratase, β- glucanase, 14–3-3 like proteins. While the susceptible genotype showed over-expression of LTP, chaperonins, and downregulation of cyanate hydratase, endochitinase, zeamatin and 14–3-3 like protein in W + E.

Over-expression of LTP and chaperonins and under-expression of 14–3-3 like proteins upon insect extract treatment were commonly observed across resistant and susceptible genotypes in W + E. Germins were differentially over-expressed in resistant genotypes in W + E treatment. Over-expression of zeamatin, endochitinase, cyanate hydratase was observed in ICSV700 while serine hydroxymethyltransferases, SOD were abundant in IS2205 in W + E treatment. The differences in over-expressed proteins in W + E in resistant genotypes suggest that they have different mechanisms to confer the resistance to the insect pest. Except for LTP and chaperonins, the susceptible genotype Swarna is not able to overexpress the genes which have a putative role in defense against the insect. The relative expression pattern of genes in early time points (3 h and 24 h) post treatment was correlated to the late (20 days after initiating *C. partellus* infestation) expression profile of proteins identified from non-targeted proteomic studies. Proteins like zeamatin, endochitinase showed a correlation in early gene expression and late protein expression pattern whereas, serine hydroxymethyltransferase, SOD, chaperonins, 14–3-3 like proteins showed a partial correlation across timepoints and genotypes. LTP and β- glucanase showed no correlation between the early gene expression and the late protein expression profile.

## Discussion

Our study originated from the observations that the two genotypes of *S. bicolor,* ICSV700 and IS2205 are resistant to insect pests while the genotype Swarna is susceptible [1]. Proteins being one of the direct effector molecules against the insects, proteomic study on these genotypes would reveal many secrets about the plant defense [41]. We carried out a comparative proteomic analysis of *S. bicolor* – *C. partellus* interaction to identify the major protein components from *S. bicolor* genotypes responsible for resistance to *C. partellus* (Fig. 1). The study was focused on 967 characterized proteins from the *S. bicolor* proteome, their analysis which allowed us to investigate the intrinsic differences in the three genotypes of *S. bicolor* and analyze their proteomic response when induced by the pest *C. partellus*. This led to the identification of several proteins that strongly supported the insect resistance traits in *S. bicolor* genotypes, and will be important for further studies.

The study revealed that the three *S. bicolor* genotypes differentially responded to the induced infestation by *C. partellus* and also had intrinsically different proteomes at steady state levels (Fig. 2A, C). Plant domestication has led to changes in the crop plant defense pathways leading to their susceptibility (as seen in the genotype Swarna) to pests and pathogens [42], while their wild relatives and improved lines (like *S. bicolor* genotypes - ICSV700, IS2205) possess the molecular components contributing to their defense [43, 44]; the proteomic analysis of these genotypes helped in discovering the protein networks involved in strengthening plant defense to insect pests.

### The differential protein complements from *S. bicolor* genotypes in response to *C. partellus*

Sixty eight proteins with differential abundance across *S. bicolor* genotypes at steady state and upon *C. partellus* infestation were identified and they were classified as significantly high or low abundance proteins (Fig. 2B; Table 2). The catalytic activities of abundant proteins were endochitinases, peroxidases and glutathione S- transferase like, all involved in promoting defense against insect pests; whereas the catalytic activities of less abundant proteins were flavone/caffeic acid 3-O-methyltransferase, ATP citrate synthase and betaine aldehyde dehydrogenase involved in the biosynthesis of a multitude of small molecules and methylated flavonoids useful in herbivore deterrence and abiotic stress [45, 46].

Cellular signaling machinery like Calmodulin-related proteins or G-protein and G protein modulators, various kinases, heat shock proteins, phenylalanine ammonia-lyase, were identified and need functional characterization to determine their contribution to *S. bicolor* pest resistance [47, 48]. Additionally, the known defense proteins like PR-5, alpha-amylase/trypsin inhibitor, osmotin, non-specific lipid transfer protein were also amongst the candidates identified, reinforcing their role in plant defense against insect pests [49, 50].

Enrichment analysis of over-represented and under-represented proteins have helped to gain a bird’s eye view of the proteome remodeling upon *C. partellus* infestation in *S. bicolor* genotypes (Fig. 3). Overall, there is more protein suppression; and selective protein accumulation as represented by the higher number of proteins in ‘response to stress’ category. The under-representation of proteins involved in translation and amino acid biosynthesis was conspicuous and as expected; but the accumulation of proteins involved in the protection and maintenance of photosynthesis upon *C. partellus* infestation, is a feature that contrasts other reports [51].

### *C. partellus* resistant *S. bicolor* genotypes have commonalities in their proteome which are not detected in the susceptible *S. bicolor* Swarna

*S. bicolor* Swarna had less abundance of proteins involved in defense, signaling and protein remodeling which might negatively influenc its defense against the invading lepidopteran pest (Fig. 2D; Supplementary Fig. 3; Supplementary Table 1). Swarna was seen to have high levels of PR proteins which are generally directed to deter pathogen attack, while the resistant *S. bicolor* genotypes are seen respond by signaling the activation of certain proteins having broad-spectrum activity against pathogens and pests or specifically directed against the pest. These are represented by proteins like chitinases, polyphenol oxidases and zeamatin.

The analysis of Pattern3 and Pattern4 proteins led to commonly expressed yet differentially abundant proteins across treatments. Serine hydroxymethyltransferase, from Pattern3, known for constitutive expression of salicylic acid-inducible genes and H_2_O_2_ detoxification genes [52] responsible for reducing the endogenous oxidative stress, was over-represented in the susceptible *S. bicolor* unlike resistant ICSV700 & IS2205 genotypes (Fig. 2D; Supplementary Table 1). It was observed in previous studies that conditions favoring oxidative stress lead to redox signaling and hormonal crosstalk responsible for fine-tuning, enhancing the defense responses in plants [53]. Further, Swarna could not accumulate proteins involved in maintaining photosynthesis upon infestation by *C. partellus* like the resistant genotypes of *S. bicolor* as represented by Pattern4. In the pair wise comparison of proteins expressed before and after infestation by *C. partellus* in the *S. bicolor* genotypes, a number of distinct proteins were identified (Fig. 3, Supplementary Data 1). Photosynthesis related proteins were strongly upregulated in ICSV700 and Swarna upon *C. partellus* infestation, however IS2205 was seen to show least perturbations as indicated by the pathway analysis (Fig. 3C). Susceptible Swarna genotype may lack networks for fine-tuning of defense responses manifested by the absence or less abundance of several proteins detected in resistant genotypes.

The insect-resistant *S. bicolor* genotypes were enriched with elongation factors and chaperons, represented by proteins 14–3-3 like proteins, calmodulins, heat shock proteins and glutamine synthetase signifying an accelerated protein synthesis, downstream signaling and refolding activity upon infestation (Fig. 4A, C; Supplementary Table 2). Similar proteomic turnover has been demonstrated recently in wheat plants as a response to the pest wheat stem sawfly [54]. 14–3-3 isoforms are differentially regulated by hormonal treatments, biotic and abiotic stress [55]; and in turn signal defense response to stresses in plants. Another protein specifically accumulated in resistant genotypes of *S. bicolor* was the superoxide dismutase (SOD), a radical quenching enzyme. High SOD activity has been noted in aphid-infested wheat plants [56], upon mite infestation in cassava [57] and has been strongly correlated to enhanced resistance to the invading pest. Differential SOD levels and isoform diversity are found to play a role in maintaining the cytosolic redox state which in turn regulates response to a variety of pathogens [58] and is probably important in mediating defense against Lepidopteran pests as well. Further, our proteomic analysis on insect-resistant *S. bicolor* indicated abundance of polyphenol oxidases (PPO) upon *C. partellus* infestation, unlike that in the susceptible genotype Swarna. Apart from its role in defense against pests and pathogens, our data supports the co-upregulation/co-expression of PPO with PSII and other photosynthesis proteins, signifying its function in protecting the photosynthetic apparatus and eventually in maintaining plant viability and growth [59]. Both the resistant genotypes at steady state (B, D) were rich in proteins involved in primary metabolic processes, efficient protein synthesis, regulation and nitrogen compound biosynthesis contributing to the insect resistance characters.

At steady-state both of the resistant *S. bicolor* genotypes were found to have a higher abundance of more than 50 proteins as compared to the susceptible genotype Swarna (Fig. 4B, C; Supplementary Table 2). These proteins were involved in maintaining a strong primary metabolism, efficient generation of energy, proficient cell communication and cell cycle in the resistant genotypes. These were represented by proteins like malate dehydrogenase which performs a key role in plant metabolism, chlorophyll a-b binding protein in photosynthesis, magnesium chelatases to regulate abscisic acid (ABA) signaling [60, 61], Glutathione S-transferases (GST) involved abiotic stress tolerance [62]. An interesting protein namely the F-box associated LRR protein was also detected only in the resistant *S. bicolor* genotypes at steady-state and may be looked upon as an important contributor to defense against insects. Recent studies have highlighted the importance of rice LRR protein as a component of plant exocyst, majorly contributing resistance to the insect pest - brown planthopper (BPH) [63].

At steady-state, ICSV700 was found to have higher levels of S-adenosyl methionine synthase (SAM synthase), subtilisin, pectinesterase, PPO, ascorbate peroxidase. Enhanced plant defense against insect pests has been demonstrated by SAM synthase through its role in polyamine synthesis [64], subtilisin, pectin esterases [65], polyphenol oxidases [66] and ascorbate peroxidase [67] showing them to be interesting candidates for reverse genetic studies and further elucidation of their mechanisms in defense (Fig. 4B and Supplementary Table 2).

### Distinctive proteomic features of *S. bicolor* genotypes

A high number of unique proteins in resistant *S. bicolor,* even at steady-state, indicated that they may act synergistically to maintain the resistance against pests, thereby, reducing the chances of infestation (Fig. 5; Table 4). Some of the high expressing unique proteins from *S. bicolor* ICSV700 at steady-state are involved in the development, maintenance of plant architecture, defense and drought tolerance represented by proliferation-associated protein 2G4, FACT complex subunit SPT16, brassinosteroid insensitive-1 like protein [68], arginine decarboxylase and nitrate reductase [69] respectively. While upon infestation by *C. partellus*, *S. bicolor* ICSV700 uniquely expressed several transcription factors and enzymes which were involved in defense against pathogens, indirect defense to herbivorous pests, development of defensive structures, wound healing /cell proliferation and showed high protein remodeling and turnover. Notable amongst them were the ATP synthase CF1 alpha subunit, β-caryophyllene synthase, and Ankyrin repeats domain-containing protein. β-Caryophyllene synthase is known to enhance the volatile emission from *S. bicolor* attracting *C. partellus*’s larval parasitoid, *Cotesia sesamiae* Cameron (Hymenoptera: Braconidae) [70]. It is exciting to detect it in infested resistant variety ICSV700 and it also explains different strategies taken by the genotypes to deter the pest. When cultivated maize varieties were not able to express β-Caryophyllene synthase upon *C. partellus* infestation, it rendered them susceptible to insect pests [71, 72]. Ankyrin repeat domain-containing proteins are involved in growth, development, protein-protein interactions and have a potential role in plant defense [73].

The other resistant variety IS2205 at steady-state uniquely expressed proteins involved in mediating stress tolerance, conferring antioxidant property and plant resistance represented by peroxidases, thiamine thiazole synthase 1, glutaredoxin and IAA amido synthase GH3, diaminopimelate decarboxylase respectively (Fig. 5; Table 4). While upon *C. partellus* infestation it uniquely expressed proteins involved in signaling stress tolerance like monogalactosyldiacyl glycerol synthase, zinc finger CCCH domain-containing protein, thiazole synthase; and proteins involved in direct defense signaling like RPP-13 like and chitinase. Maintaining thylakoid membrane biogenesis and stomata opening for retention of photosynthetic capacities in plants under stress is a prominently noted process in IS2205 *S. bicolor* genotype mediated by monogalactosyldiacyl glycerol synthase and thiazole synthase [74, 75]. Further, NBS-LRR family protein RPP-13 is an important contributor to disease, insect herbivore resistance and also abiotic stress tolerance in plants [63, 76].

In contrast to the *S. bicolor* resistant varieties the susceptible variety Swarna at steady-state uniquely expressed proteins involved in development and homeostasis and upon *C. partellus* infestation proteins for development, stress management/ defense and homeostasis represented by adenylate isopentenyltransferase, sorbitol-6-phosphate dehydrogenase, serine-threonine kinases, BOI related E3 ubiquitin-protein ligase and triacylglycerol lipase SDP1 respectively were expressed (Fig. 5 and Table 4). Serine/threonine kinases are involved in a wide array of processes ranging from signal transduction, disease resistance, developmental regulation to self- versus non-self-recognition [77] and plant defense response signaling against the pathogen [78, 79]. Ubiquitin/proteasome system (UPS) plays an important role in proteome remodeling in plant-virus interactions, defense against pathogens and survival during environmental stress [80, 81].

### The dynamics of gene expression and protein accumulation lead to differences in the correlation of gene vs proteomics profiles in *S. bicolor*

The gene expression profiles of selected genes thought to be involved in insect defense were studied in *S. bicolor* upon wounding and/or insect extract-treatment. The analysis confirmed that *S. bicolor* genotypes responded differently to the insect extract and wounding treatments. The analysis indicated that early gene expression profiles of only some gene candidates correlate with the late proteomic profiles. The differences in proteomic vs gene expression studies in *S. bicolor* can be attributed to the variation in age of plants used; field-grown vs polyhouse grow plants; actual *C. partellus* infestation vs mimicking of the infestation and prolonged infestation vs early hours after mimicking infestation in the *S. bicolor* genotypes respectively. The differences in the proteomic and mRNA expression patterns are noted in many studies and have been attributed to the existence of gene isoforms [82]; feedback regulatory circuits [83] and can be indicative of varied rates of protein translation or post-translational regulations [84].

## Conclusions

In conclusion, the proteomic analysis of 967 proteins from *S. bicolor* genotypes at steady-state and upon infestation by *C. partellus* was performed. The different statistical comparisons amongst the genotypes and treatments revealed the proteins which would be important for insect defense in *S. bicolor*. Due to the intrinsic limitations associated with protein annotations, there is a possibility of missing out on some very interesting proteins which are yet to be functionally annotated. However, the present analysis has revealed several proteins that are probably individually or synergistically used by undomesticated *S. bicolor* genotypes to strengthen its resistance to insect pests. The differentially expressed proteins in resistant vs susceptible *S. bicolor* genotypes and the uniquely expressed proteins identified, potentially contribute to the build-up of defense against *C. partellus* using different mechanisms. Further analysis of the protein-protein interactions, pathways and reverse genetic approach would help to identify the different strategies plants may adopt simultaneously to fight against insect pests and to develop agronomically beneficial yet insect-resistant crop plants.

## Supplementary Information

**Additional file 1: Supplementary Figure 1.** PCA plot across different treatment groups in *S. bicolor - C. partellus* interaction proteomics| The PCA score plot shows the variation amongst different treatments and biological and technical replicates. **Supplementary Figure 2.** GO analysis of proteins commonly expressed across resistant & susceptible *S. bicolor* upon *C. partellus* infestation| Proteins down-regulated in infested samples (highlighted in blue) and up-regulated in control samples are included in Pattern1whereas Pattern2 indicates proteins that are up-regulated in infested samples (highlighted in red) and down-regulated in control samples. GO of proteins displaying Pattern1 (38) and Pattern2 (19) are indicated molecular function (A) biological processes (B) cellular component (C). **Supplementary Figure 3.** GO biological process analysis of differentially expressed proteins across treatments in *S. bicolor* genotypes| Commonly present yet differential abundance proteins were classified into patterns based on their expression across *S. bicolor* genotypes. (A) Pattern3 listed proteins (11) generally down-regulated in *S. bicolor* upon *C. partellus* infestation, where one of the *S. bicolor* genotypes displayed a contrasting expression as indicated in the insets (difference in A/B; C/D or E/F) (B) Pattern4 listed proteins (25) generally up-regulated in *S. bicolor* upon *C. partellus* infestation, where one of the *S. bicolor* genotypes displayed a contrasting expression as indicated in the insets (difference in A/B; C/D or E/F). ‘Difference in A/B’ demonstrates a contradictory pattern in ICSV700, similarly ‘Difference in C/D’ represents in IS2205 and ‘Difference in E/F’ represents in Swarna respectively.

**Additional file 2: Supplementary Table 1.** Proteins commonly expressed in all treatments yet differentially expressed across *S. bicolor* genotypes upon *C. partellus* infestation and at steady state (Pattern 3 and Pattern 4). **Supplementary Table 2.** List of differentially abundant proteins in *S. bicolor* infested with pest *C. partellus* (A, C, E) and in *S. bicolor* at steady state (B, D, F) treatments. These proteins signify how the resistant genotypes of *S. bicolor* ICSV700 and IS2205 manifest their resistance to insect pests and the susceptible genotype Swarna cannot. **Supplementary Table 3.** Gene-specific primers used for the qRT-PCR analysis of selected candidate genes of *S. bicolor* plants induced by wounding and *C. partellus* extract. The gene expression analysis focuses on the early response (3 h to 24 h) by *S. bicolor* to the inductions.

**Additional file 3. Supplementary Data** (excel files) provided with the manuscript. 1. Sample A vs B. 2. Sample C vs D. 3. Sample E vs F. Pairwise comparison of proteins from *C. partellus* induced and steady state from the three genotypes of *S. bicolor*, ICSV700 (A vs B), IS2205 (C vs D) and Swarna (E vs F). The excels sheets provide a list of up and down regulated proteins which are obtained based on the data analysis performed with PLGS, Waters.

## Data Availability

The data in excel sheets has been attached with the manuscript as supplementary files. Any other data set generated in the study will be made available from the corresponding author on reasonable request.

## Abbreviations

JA: Jasmonic acid
SA: Salicylic acid
ROS: Reactive oxygen species
RBD: Randomized complete block design
GO: Gene ontology
