# Supplementary material for: Label-free quantitative proteomics of Sorghum bicolor reveals the proteins strengthening plant defense against insect pest Chilo partellus
Source: Proteome Sci. 2021 Apr 2;19:6. doi: 10.1186/s12953-021-00173-z (PMC8019186; doi:10.1186/s12953-021-00173-z)
Supplement: Supplementary file 1 — Additional file 1: Supplementary Figure 1. PCA plot across different treatment groups in S. bicolor - C. partellus interaction proteomics| The PCA score plot shows the variation amongst different treatments and biological and technical replicates. Supplementary Figure 2. GO analysis of proteins commonly expressed across resistant & susceptible S. bicolor upon C. partellus infestation| Proteins down-regulated in infested samples (highlighted in blue) and up-regulated in control samples are included in Pattern1whereas Pattern2 indicates proteins that are up-regulated in infested samples (highlighted in red) and down-regulated in control samples. GO of proteins displaying Pattern1 (38) and Pattern2 (19) are indicated molecular function (A) biological processes (B) cellular component (C). Supplementary Figure 3. GO biological process analysis of differentially expressed proteins across treatments in S. bicolor genotypes| Commonly present yet differential abundance proteins were classified into patterns based on their expression across S. bicolor genotypes. (A) Pattern3 listed proteins (11) generally down-regulated in S. bicolor upon C. partellus infestation, where one of the S. bicolor genotypes displayed a contrasting expression as indicated in the insets (difference in A/B; C/D or E/F) (B) Pattern4 listed proteins (25) generally up-regulated in S. bicolor upon C. partellus infestation, where one of the S. bicolor genotypes displayed a contrasting expression as indicated in the insets (difference in A/B; C/D or E/F). ‘Difference in A/B’ demonstrates a contradictory pattern in ICSV700, similarly ‘Difference in C/D’ represents in IS2205 and ‘Difference in E/F’ represents in Swarna respectively. [file 12953_2021_173_MOESM1_ESM.pptx]

## Slide 1
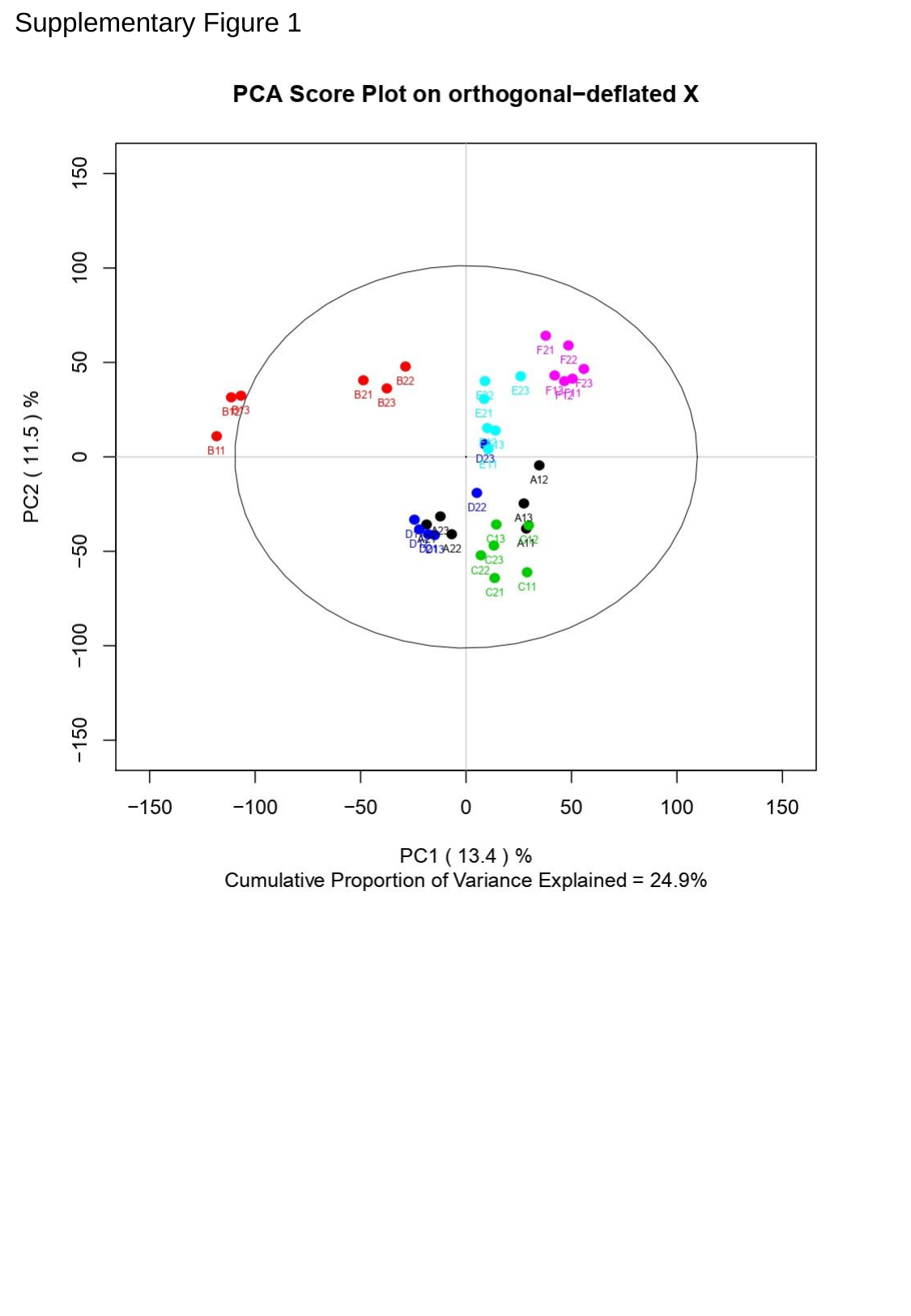

Supplementary Figure 1

## Slide 2
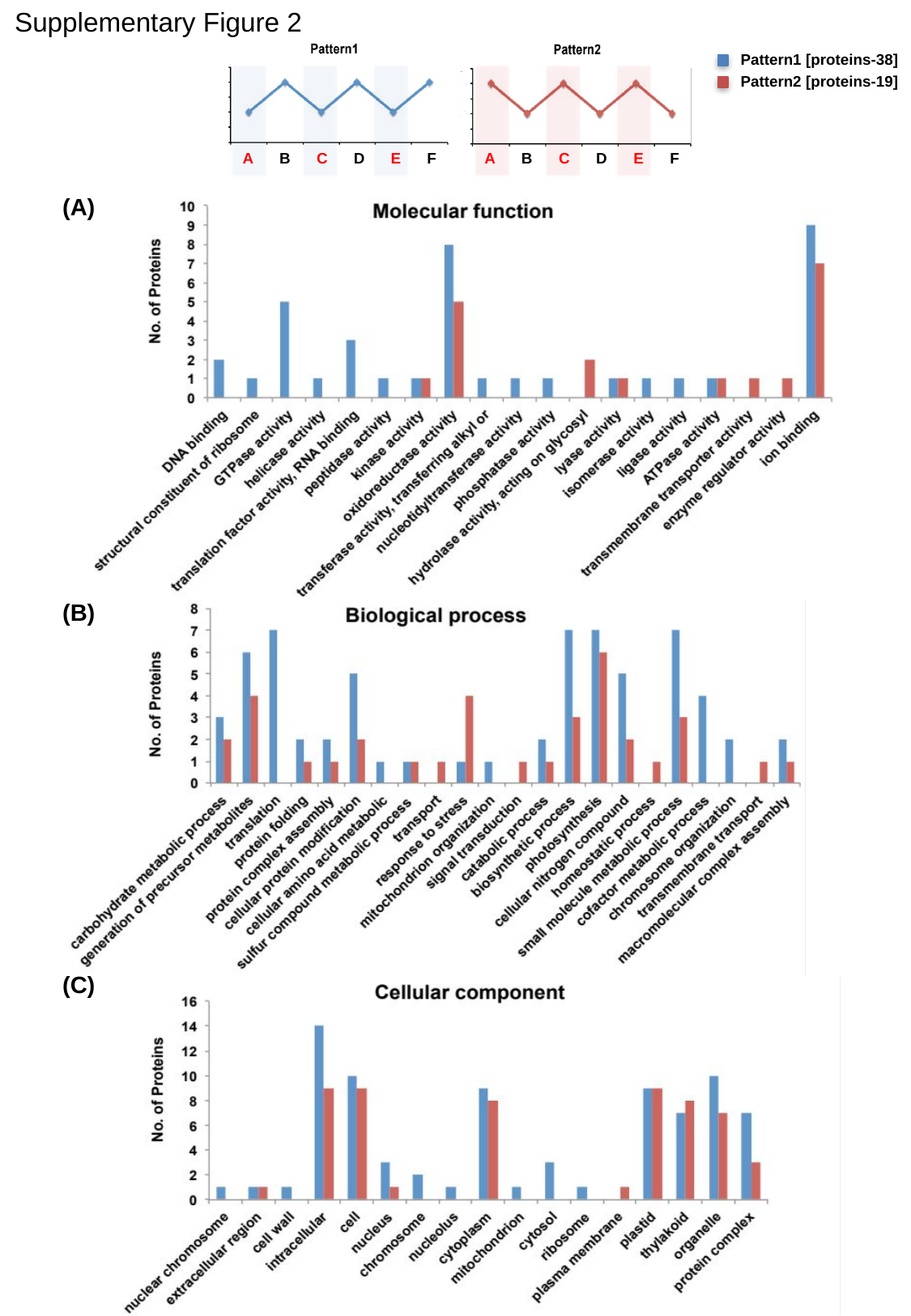

Supplementary Figure 2
A
B
C
D
E
F
A
B
C
D
E
F
Pattern1 [proteins-38]
Pattern2 [proteins-19]
(A)
(B)
(C)

## Slide 3
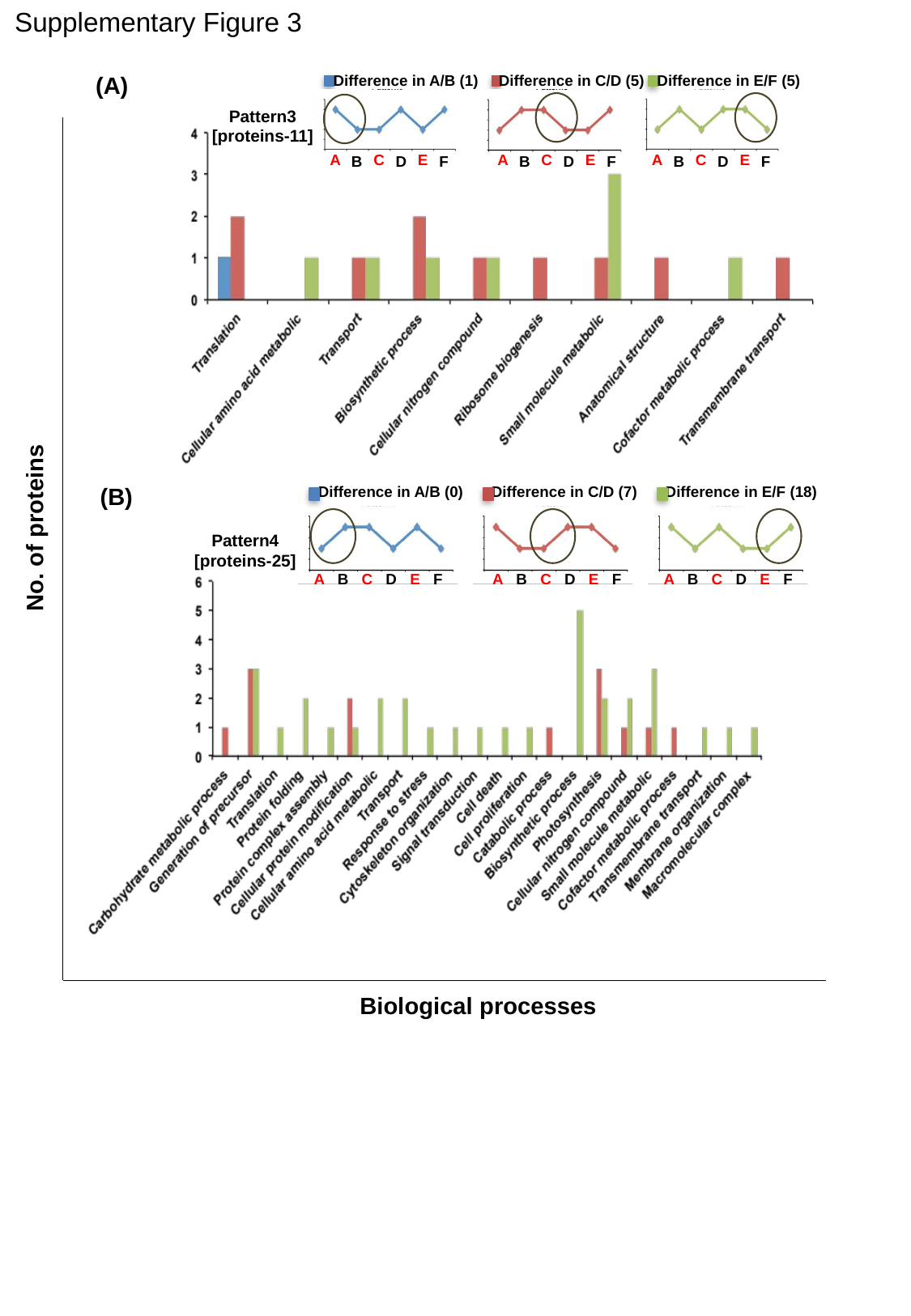

Supplementary Figure 3
(A)
 Difference in A/B (1)
A
C
E
B
D
F
 Difference in C/D (5)
A
C
E
B
D
F
 Difference in E/F (5)
A
C
E
B
D
F
Pattern3
[proteins-11]
(B)
 Difference in A/B (0)
A
B
C
D
E
F
 Difference in C/D (7)
A
B
C
D
E
F
 Difference in E/F (18)
A
B
C
D
E
F
Pattern4
[proteins-25]
No. of proteins
Biological processes
