# Supplementary material for: Label-free quantitative proteomics of Sorghum bicolor reveals the proteins strengthening plant defense against insect pest Chilo partellus
Source: Proteome Sci. 2021 Apr 2;19:6. doi: 10.1186/s12953-021-00173-z (PMC8019186; doi:10.1186/s12953-021-00173-z)
Supplement: Supplementary file 2 — Additional file 2: Supplementary Table 1. Proteins commonly expressed in all treatments yet differentially expressed across S. bicolor genotypes upon C. partellus infestation and at steady state (Pattern 3 and Pattern 4). Supplementary Table 2. List of differentially abundant proteins in S. bicolor infested with pest C. partellus (A, C, E) and in S. bicolor at steady state (B, D, F) treatments. These proteins signify how the resistant genotypes of S. bicolor ICSV700 and IS2205 manifest their resistance to insect pests and the susceptible genotype Swarna cannot. Supplementary Table 3. Gene-specific primers used for the qRT-PCR analysis of selected candidate genes of S. bicolor plants induced by wounding and C. partellus extract. The gene expression analysis focuses on the early response (3 h to 24 h) by S. bicolor to the inductions. [file 12953_2021_173_MOESM2_ESM.docx]

**Supplementary Table 1:** Proteins commonly expressed in all treatments yet differentially expressed across *S. bicolor* genotypes upon *C. partellus* infestation and at steady state (Pattern 3 and Pattern 4)

| **Pattern 3 (11)** | | | | |
| --- | --- | --- | --- | --- |
| **Protein Key** | **Protein Accession No.** | **Name** | **Classification** | **Function/Similar Protein** |
| 16961 | C5Y1T5 | similar to RuBiSCo | Pattern3 (Diff. in A/B) | Carbon fixation |
| 1 | A1E9S1 | ATP synthase subunit alpha chloroplastic OS Sorghum bicolor GN atpA PE 3 SV 1 | Pattern3 (Diff. in A/B) | Produces ATP from ADP in the presence of a proton gradient across the membrane, The alpha chain is a regulatory subunit |
| 32675 | C5WVA6 | similar to 30S ribosomal protein | Pattern3 (Diff. in C/D) | Translation |
| 26802 | C5XGT6 | ATP synthase subunit beta | Pattern3 (Diff. in C/D) | Produces ATP from ADP in the presence of a proton gradient across the membrane |
| 23912 | C5XZD9 | similar to Calcium-sensing receptor | Pattern3 (Diff. in C/D) | Calcium-sensing receptor, chloroplastic |
| 32104 | C5YMX6 | 60S acidic ribosomal protein P0 | Pattern3 (Diff. in C/D) | Translation |
| 20660 | C5XE07 | similar to Elongation factor 1-delta | Pattern3 (Diff. in C/D) | Translation elongation factor activity |
| 10349 | C5XR87 | similar to Chloroplast photosystem II 22 kDa protein | Pattern3 (Diff. in E/F) | Chloroplast photosystem II |
| 5048 | C5Z116 | similar to Germin like protein | Pattern3 (Diff. in E/F) | Resistance to broad specificity of protease, Oxalate oxidase activity |
| 17998 | C5XSH8 | similar to Fruit protein pKIWI502 | Pattern3 (Diff. in E/F) | FAD binding, Fruit protein pKIWI502 |
| 13881 | C5Y297 | Serine hydroxymethyltransferase | Pattern3 (Diff. in E/F) | 5,10-methylenetetrahydrofolate + glycine + H_2_O 🡪 tetrahydrofolate + L-serine |
| **Pattern 4 (25)** | | | | |
| **Protein Key** | **Protein Accession No.** | **Name** | **Classification** | **Function** |
| 5455 | C5X7M3 | Chlorophyll a-b binding protein | Pattern4 (Diff. in C/D) | Light-harvesting complex (LHC) functions as a light receptor |
| 9571 | C5X658 | similar to 30S ribosomal protein | Pattern4 (Diff. in C/D) | Translation |
| 24668 | C5X9I8 | similar to photosystem I reaction center subunit psaK | Pattern4 (Diff. in C/D) | Chlorophyll binding, Photosystem I reaction center 6 |
| 24119 | C5Y3L5 | similar to FBD associated F-box/LRR-repeat protein | Pattern4 (Diff. in C/D) | An integral component of membrane, responsible for protein turnover |
| 5435 | C5YVW5 | Chlorophyll a-b binding protein | Pattern4 (Diff. in C/D) | Light-harvesting complex in chloroplast |
| 14861 | C5Y5Q8 | Fructose bisphosphate aldolase | Pattern4 (Diff. in C/D) | D-fructose 1,6-bisphosphate 🡪 glycerone phosphate + D-glyceraldehyde 3-phosphate |
| 16377 | C5YVY7 | similar to DiAP3 protein | Pattern4 (Diff. in C/D) | Involved in nuclear actin polymerization |
| 15950 | C5YLG2 | Ferredoxin | Pattern4 (Diff. in E/F) | Electron carrier activity |
| 32676 | C5XF29 | similar to Cold-induced protein | Pattern4 (Diff. in E/F) | Cold-induced protein |
| 22124 | C5X9F7 | similar to Oxygen-evolving enhancer protein 2 | Pattern4 (Diff. in E/F) | Calcium ion binding, photosynthesis |
| 22636 | C5XT04 | similar to Cold shock domain protein 1 | Pattern4 (Diff. in E/F) | DNA binding, zinc ion binding, glycine-rich protein |
| 18887 | C5X4T4 | similar to Elongation factor 1-beta | Pattern4 (Diff. in E/F) | Translation elongation factor activity |
| 75 | A1E9X5 | Photosystem I iron-sulfur center | Pattern4 (Diff. in E/F) | Reduced plastocyanin + oxidized ferredoxin + light 🡪 oxidized plastocyanin + reduced ferredoxin |
| 15352 | C5YJN5 | similar to NADPH quinone oxidoreductase subunit S | Pattern4 (Diff. in E/F) | Photosynthetic electron transport chain, NAD(P)H-quinone oxidoreductase subunit S, chloroplastic |
| 9747 | C5YRL2 | Nonspecific lipid transfer protein | Pattern4 (Diff. in E/F) | Defense against bacteria & fungi |
| 20404 | C5Z4B6 | Profilin | Pattern4 (Diff. in E/F) | Affects the structure of the cytoskeleton. At high concentrations prevents the polymerization of actin, whereas it enhances it at low concentrations, accumulation at the site of infection |
| 11513 | C5WU61 | similar to Chloroplast post-illumination chlorophyll fluorescence increase protein | Pattern4 (Diff. in E/F) | Chloroplast post-illumination chlorophyll fluorescence increase protein |
| 20221 | C5YKK8 | similar to peptidyl-propyl cis-trans isomerase | Pattern4 (Diff. in E/F) | Role in plant defense against bacteria |
| 2 | A1E9T1 | ATP synthase subunit beta chloroplastic | Pattern4 (Diff. in E/F) | Produces ATP from ADP in the presence of a proton gradient across the membrane |
| 30530 | C5XRL7 | similar to Translationally-controlled tumor protein-like protein | Pattern4 (Diff. in E/F) | Calcium ion binding, prevents apoptosis |
| 14592 | C5XXB8 | GrpE protein homolog | Pattern4 (Diff. in E/F) | Component of the PAM complex, a complex required for the translocation of transit peptide-containing proteins from the inner membrane into the mitochondrial matrix in an ATP-dependent manner, associated with HSP70 |
| 811 | C5XFP1 | Cysteine synthase | Pattern4 (Diff. in E/F) | O-acetyl-L-serine + hydrogen sulfide 🡪 L-cysteine + acetate, Pyridoxal 5'-phosphate cofactor |
| 31871 | C5X5U3 | similar to 28 kDa ribonucleoprotein | Pattern4 (Diff. in E/F) | Nucleic acid-binding protein 1 |
| 2322 | C5YN49 | Superoxide dismutase Cu Zn | Pattern4 (Diff. in E/F) | Destroys radicals which are normally produced within the cells and which are toxic to biological systems |
| 5345 | C5WVT9 | similar to G protein alpha subunit | Pattern4 (Diff. in E/F) | GTPase activity, G protein-coupled receptor signaling pathway |

**Supplementary Table 2:** List of differentially abundant proteins in *S. bicolor* infested with pest *C. partellus* (A, C, E) and in *S. bicolor* at steady state (B, D, F) treatments. These proteins signify how the resistant genotypes of *S. bicolor* ICSV700 and IS2205 manifest their resistance to insect pests and the susceptible genotype Swarna cannot.

| **Comparison of proteins in *S. bicolor* varieties upon treatment (A, C, E)** | | | |
| --- | --- | --- | --- |
| **Protein Key** | **Protein Accession No.** | **Name** | **Function** |
| **Proteins up in A, C and down in E** | | | |
| 2387 | C5XHR8 | similar to β-1,3-glucanase A, Pathogenesis-related protein 6 | Glycoside hydrolase family, Hydrolysis of O-glycosyl compounds, Carbohydrate metabolic process |
| 20404 | C5Z4B6 | Profilin | Affects the structure of the cytoskeleton. At high concentrations prevents the polymerization of actin, whereas it enhances it at low concentrations, accumulation at the site of infection |
| 19011 | C5XFC3 | similar to chitinase | Chitin degradation |
| 15352 | C5YJN5 | similar to NADPH quinone oxidoreductase subunit S | Photosynthetic electron transport chain, NAD(P)H-quinone oxidoreductase subunit S, chloroplastic |
| 2182 | C5YIC3 | similar to Polyphenol oxidase family protein | Catechol oxidase activity, 2 catechol + O_2_ 🡪 2 1,2-benzoquinone + 2 H_2_O |
| 18887 | C5X4T4 | similar to Elongation factor 1-beta | Translation elongation factor activity |
| 23791 | C5Y3X7 | similar to Dynein heavy chain-like protein, Aminotransferase like | Upregulated in tobacco on TMV infection |
| 17003 | C5Y9W4 | similar to 14-3-3-like protein GF14-6 | Protein domain-specific binding |
| 22636 | C5XT04 | similar to Cold shock domain protein 1 | DNA binding, zinc ion binding, glycine-rich protein |
| 15950 | C5YLG2 | Ferredoxin | Electron carrier activity |
| 13253 | C5XR31 | similar to Aldo/keto reductase family protein | Aldo-keto reductase family |
| 73 | Q9T2L6 | Photosystem I P700 chlorophyll a apoprotein A1 | Metal ion binding in PSI |
| 13048 | C5YDD1 | similar to Transketolase, chloroplastic | Transketolase activity, photoprotection, involved in isoprenoid formation |
| 9254 | C5XCE2 | similar to Zeamatin-like protein | Inhibition of trypsin and α-amylases |
| 5724 | C5X768 | similar to Pyridoxine biosynthesis protein ER1 | Pyridoxal phosphate biosynthesis, a cofactor of ACC deaminase & ACC synthase |
| 372 | C5X1U2 | similar to CaM protein | Calcium ion binding & signaling, regulation of gene expression |
| 11513 | C5WU61 | similar to Chloroplast post-illumination chlorophyll fluorescence increase protein | Chloroplast post-illumination chlorophyll fluorescence increase protein |
| 28879 | C5WRV5 | similar to RuBisCO large subunit-binding protein subunit alpha | Chaperonin Cpn60/TCP-1 family |
| 15444 | C5XJT8 | similar to Protein disulfide isomerase-like 2-2 | Induced in response to the fungal pathogen |
| 2322 | C5YN49 | Superoxide dismutase Cu Zn OS Sorghum bicolor GN Sb07g023950 PE 3 SV 1 | Destroys radicals which are normally produced within the cells and which are toxic to biological systems, 2 superoxides + 2 H^+^ 🡪 O_2_ + H_2_O_2_ |
| 6977 | C5Z0B5 | Phosphoglycerate kinase | ATP + 3-phospho-D-glycerate 🡪 ADP + 3-phospho-D-glyceroyl phosphate |
| 399 | B5B9V8 | Glutamine synthetase | ATP + L-glutamate + NH_3_ 🡪 ADP + phosphate + L-glutamine, role in NO signaling |
| 30151 | C5Z8N5 | similar to EG-45 like domain-containing protein | Chitinase activity |
| 4680 | C5XBK5 | Elongation factor 1 alpha | GTPase activity |
| 11895 | C5YSV2 | similar to Thaumatin-like protein, Pathogenesis-related protein 5 | Thaumatin |
| 78 | A1E9Q4 | Photosystem II protein D1 | 2 H_2_O + 2 plastoquinone + 4 light 🡪 O_2_ + 2 plastoquinol |
| 20660 | C5XE07 | similar to Elongation factor 1-delta | Translation elongation factor activity |
| 32612 | C5Z2S4 | similar to TCP-1/cpn60 chaperonin family protein isoform 1 | Chaperonin Cpn60/TCP-1 family |
| 26556 | C5YW53 | similar to 60 kDa chaperonin alpha subunit | Chaperonin Cpn60/TCP-1 family |
| **Proteins down in A, C and up in E** | | | |
| 26780 | C5XIL2 | similar to NAD(P)H-quinone oxidoreductase subunit O, chloroplastic | NADPH-quinone oxidoreductase |
| 27365 | C5X972 | Nucleoside diphosphate kinase | Response to bacterial infection |
| 1320 | C5YUB8 | similar to Stem 28 kDa glycoprotein | Acid phosphatase activity |
| 9824 | C5YIU7 | similar to Thylakoidal lumenal 19 kDa protein | Calcium ion binding, photosynthesis |
| 8412 | C5X7N2 | Chlorophyll a-b binding protein, chloroplastic | Chlorophyll a-b binding protein |
| 26814 | C5X0W1 | similar to Chloroplast oxygen-evolving complex/thylakoid lumenal 25.6kDa protein | Calcium ion binding, Photosynthesis |
| 24171 | C5X7Q2 | similar to 1-phosphatidylinositol-4-phosphate 5-kinase | GTPase activity, UDP-glucose metabolic process, alpha-D-glucose 1-phosphate + UTP 🡪 diphosphate + UDP-D-glucose |
| **Other differentially regulated proteins** | | | |
| 16377 | C5YVY7 | similar to DiAP3 protein | Involved in nuclear actin polymerization |
| 9571 | C5X658 | similar to 30S ribosomal protein | Translation |
| 24548 | C5WRH6 | similar to Peptide methionine sulfoxide reductase A4, chloroplastic | Peptide-methionine(S)-S-oxide reductase |
| 28788 | C5WQE1 | similar to Protein P21, α-amylase/ trypsin inhibitor | Inhibits the activity of CDK proteins |
| 10349 | C5XR87 | similar to Chloroplast photosystem II 22 kDa protein | Chloroplast photosystem II, involved in light stress response |
| 656 | C5YSK7 | similar to Pathogenesis-related protein 5 | Thaumatin protein family |
| 9747 | C5YRL2 | Nonspecific lipid transfer protein | Transfer phospholipids as well as galactolipids across membranes |
| 21686 | C5XWZ5 | Putative uncharacterized protein Sb04g005860 OS Sorghum bicolor GN Sb04g005860 PE 4 SV 1 | Cyclase protein |
| 74 | A1E9S3 | Photosystem I P700 chlorophyll a apoprotein A2 | Reduced plastocyanin + oxidized ferredoxin + light 🡪 oxidized plastocyanin + reduced ferredoxin |
| 9604 | C5Z469 | Peroxidase | Oxidative stress, 2 phenolic donor + H_2_O_2_ 🡪 2 phenoxyl radical of the donor + 2 H_2_O |
| 80 | A1E9R0 | Photosystem II CP43 reaction center protein | Components of the core complex of PSII |
| 22124 | C5X9F7 | similar to Oxygen-evolving enhancer protein 2 | Calcium ion binding, photosynthesis |
| 20517 | C5XJW7 | similar to ATP synthase CF1 beta subunit | Cell death regulator, lower extracellular ATP induces death |
| 18171 | C5YJQ8 | similar to 14-3-3-like protein A | Protein domain-specific binding |
| 26240 | C5YHK9 | similar to 14-3-3-like protein A | Protein domain-specific binding |
| 2 | A1E9T1 | ATP synthase subunit beta chloroplastic | Produces ATP from ADP in the presence of a proton gradient across the membrane. The catalytic sites are hosted primarily by the beta subunits |
| 32104 | C5YMX6 | 60S acidic ribosomal protein P0 | Ribosome biogenesis |
| 10231 | C5XWZ7 | similar to Cyclase | Tryptophan catabolic process to kynurenine, production of ROS, NOS |
| 389 | Q4VQB2 | Pathogenesis-related protein 10b | Associated with hypersensitive cell death response |
| 24967 | C5YZJ4 | S adenosylmethionine synthase | Involved in ethylene biosynthesis |
| 811 | C5XFP1 | Cysteine synthase | O-acetyl-L-serine + hydrogen sulfide 🡪 L-cysteine + acetate |
| 25305 | C5Z6H7 | similar to Calvin cycle protein CP12-1, chloroplastic | Chaperone of GAPDH & other enzymes in the Calvin cycle, prevention from oxidative stress |
| 32676 | C5XF29 | similar to Cold-induced protein | Cold-induced protein, involved in ABA biosynthesis |
| 16961 | C5Y1T5 | similar to RuBiSCo | ATP binding |
| 14789 | C5YYX3 | similar to Dehydroascorbate reductase | Glutathione transferase activity |
| 32675 | C5WVA6 | similar to 30S ribosomal protein | Ribosomal protein S5 |
| 36 | A1E9T7 | Cytochrome f | Electron transfer between PSII & PSI |
| 31118 | C5XC52 | similar to Cinnamyl alcohol dehydrogenase family | Cinnamyl alcohol dehydrogenase |
| 5048 | C5Z116 | similar to Germin like protein | Nutrient reservoir activity |
| 21271 | C5XQ07 | similar to Triosephosphate isomerase | Glycolytic process |
| 28696 | C5WZ31 | similar to Photosystem I reaction center subunit III | Photosystem I, reaction center subunit III |
| 26971 | C5X8S2 | similar to Pathogenesis-related protein-1 | Cysteine-rich secretory protein, allergen V5/Tpx-1 |
| 19466 | C5Z269 | similar to Ferredoxin-NADP reductase | Ferredoxin-NADP reductase |
| 17998 | C5XSH8 | similar to Fruit protein pKIWI502 | FAD binding, Fruit protein pKIWI502 |
| 26619 | C5Y817 | similar to Carboxyl terminal peptidase precursor | Peptidase activity |
| 3577 | C5XN16 | similar to Glutathione S transferase GSTU6 | Glutathione transferase activity, toxin catabolic process |
| **Comparison of proteins in *S. bicolor* varieties at steady state (B, D, F)** | | | |
| **Protein Key** | **Protein Accession No.** | **Name** | **Function** |
| **Proteins up in B, D and down in F** | | | |
| 10349 | C5XR87 | similar to Photosystem II 22 kDa protein | Chlorophyll a-b binding protein |
| 26240 | C5YHK9 | similar to 14-3-3 like protein | Protein domain-specific binding |
| 19234 | C5Z529 | similar to Glutathione S-transferase | Elongation factor activity |
| 25790 | C5XUF6 | similar to Leucine aminopeptidase 2 | Exopeptidase activity, leucine aminopeptidase |
| 17391 | C5Z5R1 | similar to Fructose-bisphosphate aldolase | Glycolytic process |
| 7601 | C5X4C8 | similar to 60S ribosomal protein L8 | Ribosomal protein L2 |
| 31631 | C5WXA8 | similar to Protochlorophyllide reductase B | Protochlorophyllide reductase activity. Involved in light harvesting |
| 16609 | C5WYF2 | Malate dehydrogenase | (S)-malate + NAD^+^ 🡪 oxaloacetate + NADH |
| 10950 | C5YF84 | similar to Chaperone protein ClpC1, chloroplastic | ATP binding |
| 2408 | C5YW13 | Ketol-acid reductoisomerase | Acetolactate forming, involved in isoleucine-valine biosynthesis |
| 267 | C5WTL6 | Histone H4 | DNA binding |
| 14861 | C5Y5Q8 | Fructose bisphosphate aldolase | D-fructose 1,6-bisphosphate 🡪 glycerone phosphate + D-glyceraldehyde 3-phosphate |
| 5455 | C5X7M3 | Chlorophyll a-b binding protein, chloroplastic | Photosynthesis, light harvesting |
| 17003 | C5Y9W4 | similar to 14-3-3-like protein GF14-6 | Protein domain-specific binding |
| 969 | C5YMV5 | similar to Malate dehydrogenase [NADP] 1, chloroplastic | Malate dehydrogenase type 2, NADP dependent |
| 23791 | C5Y3X7 | similar to Dynein heavy chain-like protein, Aminotransferase like | Up-regulated in tobacco on TMV infection |
| 28193 | C5X1V3 | similar to 30S ribosomal protein S1 | mRNA binding, response to cytokinin |
| 28508 | C5YMW4 | similar to Chaperone protein ClpC4 | Protein folding |
| 24119 | C5Y3L5 | similar to FBD associated F-box/LRR-repeat protein | Protein binding |
| 28345 | C5XKV8 | similar to Glutathione S-transferase GSTF2 | Glutathione transferase activity |
| 6649 | C5XVM4 | similar to Histone H3 | Histone H3 |
| 6776 | C5X4S1 | Elongation factor Ts | Translation elongation factor activity |
| 5834 | C5YPZ1 | similar to Heat shock protein | Heat shock protein family 7, ATP binding |
| 31706 | C5X7L1 | similar to 60S ribosomal protein L14 | Structural constituent of ribosome |
| 25758 | C5YJ75 | similar to Heat shock protein 90 | Heat shock protein Hsp90 family |
| 15924 | C5XXS0 | Succinate CoA ligase subunit beta | Carbohydrate metabolism |
| 32336 | C5X0U5 | [similar to Photosystem II 11 kDa protein](https://blast.ncbi.nlm.nih.gov/Blast.cgi#alnHdr_514813301) | Assembly of PSII |
| 18171 | C5YJQ8 | similar to 14-3-3-like protein A | Protein domain-specific binding |
| 26360 | C5WX74 | similar to 60S ribosomal protein L4 | Ribosomal protein L4/L1e |
| 74 | A1E9S3 | Photosystem I P700 chlorophyll a apoprotein A2 | Reduced plastocyanin + oxidized ferredoxin + light 🡪 oxidized plastocyanin + reduced ferredoxin |
| 1374 | C5XWJ8 | Elongation factor Tu | GTPase activity |
| 17625 | C5WPF7 | similar to Heat shock cognate 70 kDa protein 2 | Heat shock protein 70 family, Response to the bacterium |
| 17998 | C5XSH8 | similar to Fruit protein pKIWI502 | FAD binding, oxidoreductase activity |
| 31557 | C5YH46 | similar to 40S ribosomal protein S19 | Ribosomal protein S19e |
| 31636 | C5X0P2 | similar to 60S ribosomal protein L22-2 | Ribosomal protein L22e |
| 36 | A1E9T7 | Cytochrome f | Electron transfer between PSII & PSI |
| 29257 | C5X0W2 | similar to 50S ribosomal protein L11 | Ribosomal protein L11/L12 |
| 80 | A1E9R0 | Photosystem II CP43 reaction center protein | Cyclic electron transporter, a component of the core complex of PSII |
| 24314 | C5YU98 | Malic enzyme | Oxidoreductase activity |
| 25839 | C5X3T9 | similar to Heat shock protein | Heat shock protein Hsp90 family |
| 5435 | C5YVW5 | Chlorophyll a-b binding protein, chloroplastic | Photosynthesis, light harvesting |
| 18642 | C5XSW9 | similar to Inorganic pyrophosphatase | Involved in response to biotic stress |
| 2164 | C5Y7U2 | similar to Chlorophyll a-b binding protein, chloroplastic | Chlorophyll a-b binding protein |
| 9025 | C5YCZ2 | Elongation factor G chloroplastic | Elongation factor activity in plastids |
| 28815 | C5WN47 | similar to Luminal-binding protein 4 | Heat shock protein 70 family |
| 14866 | C5YSP7 | Fructose-bisphosphate aldolase | Glycolytic process |
| 30199 | C5YSM6 | Mg-protoporphyrin IX chelatase | Chlorophyll biosynthesis |
| 2872 | C5WTC1 | Chlorophyll a-b binding protein, chloroplastic | Photosynthesis, light harvesting |
| **Proteins down in B, D and up in F** | | | |
| 23741 | C5YCJ6 | similar to Ferredoxin/Thioredoxin reductase, variable chain | ferredoxin-thioredoxin reductase |
| 27041 | C5XDP0 | similar to 20 kDa chaperonin | GroES Chaperonin family, a positive regulator of SODs |
| 15352 | C5YJN5 | [similar to NAD(P)H-quinone oxidoreductase subunit S, chloroplastic](https://blast.ncbi.nlm.nih.gov/Blast.cgi#alnHdr_514813301) | Photosynthetic electron transport chain |
| **Other differentially regulated proteins** | | | |
| 16961 | C5Y1T5 | similar to RuBiSCo | ATP binding |
| 30530 | C5XRL7 | similar to Translationally-controlled tumor protein-like protein | Mss-4 like superfamily |
| 25741 | C5XA61 | similar to ATP/GTP binding protein | Protein serine/threonine kinase activity, bifunctional kinase-phosphorylase |
| 9571 | C5X658 | similar to 30S ribosomal protein S31 | Translation |
| 9747 | C5YRL2 | Nonspecific lipid transfer protein | Transfer phospholipids as well as galactolipids across membranes |
| 22227 | C5X0G5 | similar to Cell division control protein 48-like protein E | AAA ATPase CDC48 family |
| 32676 | C5XF29 | similar to Cold-induced protein | Cold-induced protein, Nodulin related protein |
| 20221 | C5YKK8 | similar to Peptidyl-prolyl cis-trans isomerase | Interconversion of cis-trans isomers of peptide bond |
| 14592 | C5XXB8 | GrpE protein homolog | Stimulates the hydrolysis and exchange of adenyl nucleotides by other proteins |
| 2387 | C5XHR8 | similar to Beta-1,3-glucanase A | Hydrolysis of O-glycosyl compounds, Carbohydrate metabolic process |
| 6773 | C6JSG0 | similar to Translation elongation factor family protein | GTPase activity |
| 866 | C5XJZ3 | similar to Elongation factor 2 | GTPase activity |
| 9877 | C5Y596 | similar to Subtilisin like serine endopeptidase family protein | Subtilisin like serine endopeptidase family protein |
| 24967 | C5YZJ4 | S adenosylmethionine synthase | ATP + L-methionine + H2O 🡪 phosphate + diphosphate + S-adenosyl-L-methionine, ethylene biosynthesis |
| 21020 | C5Z8X0 | similar to Eukaryotic initiation factor 4A | Translation initiation factor IF4a |
| 32065 | C5YLK6 | similar to NADP-dependent glyceraldehyde-3-phosphate dehydrogenase | Aldehyde dehydrogenase activity |
| 2182 | C5YIC3 | similar to Polyphenol oxidase family protein | Catechol oxidase activity, 2 catechol + O_2_ 🡪 2 1,2-benzoquinone + 2 H_2_O |
| 167 | Q84LQ5 | Malic enzyme | Malate dehydrogenase activity, induced upon wounding |
| 30618 | C5WZ87 | similar to Ribosomal protein S9 | Structural constituent of ribosome |
| 79 | A1E9V0 | Photosystem II CP47 reaction center protein | Electron transporter |
| 26802 | C5XGT6 | similar to ATP synthase subunit beta | Cell death regulator, lower extracellular ATP induces death |
| 27747 | C5X1K7 | similar to GDP-mannose 3,5-epimerase 1 | GDP-mannose-3,5-epimerase activity, involved in the synthesis of ascorbic acid which is involved in defense regulation |
| 20857 | C5WLV9 | Glucose 1 phosphate adenylyltransferase | ATP + alpha-D-glucose 1-phosphate 🡪 diphosphate + ADP-glucose |
| 102 | A1E9W6 | 50S ribosomal protein L2 chloroplastic | RNA binding, transferase activity |
| 52 | A1E9Y0 | NADPH quinone oxidoreductase subunit H chloroplastic | Quinone binding, NAD(P)H + plastoquinone 🡪 NAD(P)^+^ + plastoquinol |
| 23353 | C5XW45 | Glyceraldehyde 3 phosphate dehydrogenase | Oxidoreductase activity, Glucose metabolic process |
| 28031 | C5XIT6 | Pectinesterase | Pectin + n H_2_O 🡪 n methanol + pectate |
| 21506 | C5XS12 | similar to 50S ribosomal protein L19, chloroplastic | Translation |
| 31796 | C5Y8Q1 | similar to Ascorbate peroxidase | Cytochrome c-peroxidase activity, Response to reactive oxygen species |

**Supplementary Table 3:** Gene-specific primers used for the qRT-PCR analysis of selected candidate genes of *S. bicolor* plants induced by wounding and *C. partellus* extract. The gene expression analysis focuses on the early response (3 h to 24 h) by *S. bicolor* to the inductions.

| **Protein_AccnNo** | **Protein Name** | **NCBI Reference No** | **F/ R** | **Primer Sequence (5’-3’)** | **Tm** | **% GC** | **Product Size (bp)** |
| --- | --- | --- | --- | --- | --- | --- | --- |
| C5WM27 | Tubulin alpha chain | XM_002466490.1 | F | AGTGTCCTGTCCACCCACTC | 60.01 | 60 | 222 |
|  |  |  | R | CACCAGGTTGGTCTGGAACT | 60 | 55 |  |
| C5Y297 | Serine hydroxymethyl transferase | XM_002449419.2 | F | AGGGCTGGCATGATCTTCTA | 59.8 | 50 | 201 |
|  |  |  | R | CTTTGCATAGGCCTTGAAGC | 59.98 | 50 |  |
| C5Z116 | Germin | XM_002439305.2 | F | CACCGCCAACGACTTCTACT | 60.31 | 55 | 273 |
|  |  |  | R | GCGTACAGCGTCTTGGTGTA | 59.94 | 55 |  |
| C5WYI7 | Cyanate hydratase | XM_002467053.2 | F | TGATGGAGGTGAAGGAGGAG | 60.19 | 55 | 181 |
|  |  |  | R | TGCATCATGAGTTGGACGAG | 60.84 | 50 |  |
| C5XHR8 | β-glucanases | XM_002459029.2 | F | CCTACAGGAGTGCAAGCCAT | 59.75 | 55 | 250 |
|  |  |  | R | GCACGTTGCTCTTCACCC | 59.05 | 61 |  |
| C5YRL0 | Lipid transfer protein | XM_002442729.2 | F | CAGGTGAACTCCGCGATTAG | 58.44 | 55 | 233 |
|  |  |  | R | ATGGTGTAGGGGATGTTGACG | 59.79 | 52 |  |
| C5XCE2 | Zeamatin | XM_002463032.2 | F | CGTGTTCAAGCAGGACGTCTA | 60.34 | 52 | 122 |
|  |  |  | R | CGTCGTCCTTTGGGTAGCTG | 60.74 | 60 |  |
| C5XFC3 | Endochitinase | XM_002456038.2 | F | TCAAGTGGGCTCCTGTTCTG | 59.6 | 55 | 159 |
|  |  |  | R | GGAACGCCAGGATGACGTAG | 60.53 | 60 |  |
| C5YN49 | Superoxide dismutase | XM_002445626.2 | F | CGGGTGACCTGGGAAACATT | 60.25 | 55 | 213 |
|  |  |  | R | TCAGGCCAACAACACCACAT | 60.11 | 50 |  |
| C5YW53 | Chaperonin | XM_002440842.2 | F | ACCTGATTGAGGCTGGTGTG | 59.96 | 55 | 126 |
|  |  |  | R | GCTTCGGCTTTGGCTTTTCA | 59.97 | 50 |  |
| C5Y9W4 | 14-3-3 like protein | XM_002446581.2 | F | ATTGCTTTGGCTGAGCTTGC | 60.04 | 50 | 154 |
|  |  |  | R | GGGTGTCCAGTTCCGAGATG | 60.11 | 60 |  |
